# *Casuarina glauca* branchlets’ extract as a potential treatment for ulcerative colitis: chemical composition, *in silico* and *in vivo* studies

**DOI:** 10.3389/fphar.2023.1322181

**Published:** 2023-12-22

**Authors:** Maged E. Mohamed, Azza M. El-Shafae, Eman Fikry, Samar S. Elbaramawi, Mahmoud H. Elbatreek, Nora Tawfeek

**Affiliations:** ^1^ Department of Pharmaceutical Sciences, College of Clinical Pharmacy, King Faisal University, Al-Ahsa, Saudi Arabia; ^2^ Department of Pharmacognosy, Faculty of Pharmacy, Zagazig University, Zagazig, Egypt; ^3^ Department of Medicinal Chemistry, Faculty of Pharmacy, Zagazig University, Zagazig, Egypt; ^4^ Department of Pharmacology and Toxicology, Faculty of Pharmacy, Zagazig University, Zagazig, Egypt

**Keywords:** *Casuarina glauca*, ulcerative colitis, network pharmacology, docking, LRRK2, EGFR

## Abstract

Ulcerative colitis (UC) is an inflammatory bowel disease that is often resistant to current treatment options, leading to a need for alternative therapies. Herbal products have shown promise in managing various conditions, including UC. However, the potential of *Casuarina glauca* branchlets ethanolic extract (CGBRE) in treating UC has not been explored. This study aimed to analyze the chemical composition of CGBRE and evaluate its efficacy in UC treatment through *in silico* and *in vivo* experiments. LC-ESI-MS/MS was used to identify 86 compounds in CGBRE, with 21 potential bioactive compounds determined through pharmacokinetic analysis. Network pharmacology analysis revealed 171 potential UC targets for the bioactive compounds, including EGFR, LRRK2, and HSP90 as top targets, which were found to bind to key CGBRE compounds through molecular docking. Molecular docking findings suggested that CGBRE may be effective in the prevention or treatment of ulcerative colitis mediated by these proteins, where key CGBRE compounds exhibited good binding affinities through formation of numerous interactions. *In vivo* studies in rats with acetic acid-induced UC demonstrated that oral administration of 300 mg/kg CGBRE for 6 days reduced UC symptoms and colonic expression of EGFR, LRRK2, and HSP90. These findings supported the therapeutic potential of CGBRE in UC and suggested the need for further preclinical and clinical investigation.

## 1 Introduction

Ulcerative colitis (UC) is a chronic progressive inflammatory ailment that targets the colon and rectum. This disease burden is expanding globally and associated with increased risk of cancer and poor quality of life (Alatab et al., 2020). The pathophysiology of UC is intricate and not yet fully comprehended. It is characterized by abnormal immune response, heightened inflammation, and microbial dysbiosis leading to intestinal barrier dysfunction and ultimately disruption (Kobayashi et al., 2020). Conventional therapy for UC is only symptom-focused and many patients are unresponsive or intolerant to such medications (Matsuoka and Hibi, 2023). New advanced therapies have shown enhanced efficacy, yet still ineffective in some patients with a remission rate of less than 50%. Additionally, a significant number of drugs exhibit a secondary loss of response. Eventually around one-fourth of UC patients require surgical intervention (Samuel et al., 2013; Peyrin-Biroulet et al., 2019; Kobayashi et al., 2020; Cai et al., 2021).

Herbal products are commonly used for management of various conditions, and some have shown promising outcomes in UC (Tang et al., 2011; Ben-Horin et al., 2022; Gupta et al., 2022). Casuarinaceae is a family of angiosperms assigned to the order Fagales and comprises about 96 species of multipurpose trees and shrubs. Species of Casuarinaceae are commonly named as she-oak, bull-oak, ironwood, beefwood, or Australian pine and are naturally growing in Australia, Southeast Asia, Malaysia, and the Polynesian and Melanesian regions of the Pacific (Wilson and Johnson, 1989; Diouf et al., 2008; Christenhusz and Byng, 2016). Morphologically, they are characterized by a distinctive conifer like appearance where the leaves are reduced into tiny, lanceolate, scale-like leaves, 1 mm in length, surrounding green, jointed, needle-like branchlets (Diouf et al., 2008). Formerly, species of this family were originally incorporated in a single genus, *Casurina*. However, based on morphological and chromosomal evidence, it was recently split into four distinct genera, including *Gymnostoma* L.A.S. Johnson, *Ceuthostoma* L.A.S. Johnson, *Casuarina* L., and *Allocasuarina* L.A.S. Johnson (Sogo et al., 2001; Diouf et al., 2008).

Genus *Casuarina* includes 17 species of woody trees that extended thoroughly in tropics, subtropics as well as Mediterranean countries owing to their ability to adapt climate changes and different environmental conditions, in addition to their fast growth and atmospheric nitrogen fixation capacity (Kulkarni, 2019). The name *Casuarina* originated from the Papuan word “kasuari” which describes the resemblance between the drooping tree foliage and the Cassowary bird feathers (Diouf et al., 2008). Plants from genus *Casuarina* were reported to contain various constituents from different chemical classes such as flavonoids, phenolics, alkaloids, tannins, terpenoids, diarylheptanoids, steroids and saponins (Kaneda et al., 1990; Takahashi et al., 1999; Zhang et al., 2010; Rahman, 2012; Al-Snafi, 2015; Gopichand et al., 2015; Pawar and Nasreen, 2018; Muthuraj et al., 2020; Wang et al., 2020; Xu et al., 2022). From a pharmacological perspective, *C. equisetifolia* and its hybrids have garnered significant attention due to their rapid growth and visually appealing dense crown. As a result, these species are predominantly cultivated as ornamental plants (Chonglu et al., 2015), which may account for the prevalence of pharmacological investigations conducted on *Casuarina equisetifolia*. Traditionally, *Casuarina* species, specifically *C. equisetifolia*, have been utilized for the management of gastrointestinal ailments such as constipation, diarrhea, dysentery, and stomach ulcers. Additionally, the bark of *C. equisetifolia* was employed as an astringent for the treatment of stomachaches, diarrhea, and dysentery, while the leaves were utilized as an antispasmodic for colic and the seeds as an antispasmodic (Al-Snafi, 2015). The methanolic extract of the bark of this plant demonstrated antispasmodic activity by reducing ACh, histamine, KCl, and BaCl_2_ induced contractions in isolated ileum, and enhancing the effect of nifedipine. These findings suggested that the extract possesses antimuscarinic and antihistaminic activities, and acts as a calcium channel blocker (Rahman, 2012). Similarly, the ethanolic extract of the same plant exhibited anti-ulcer and gastroprotective properties in Albino rats through induced gastric ulcer models involving ethanol, indomethacin, and cold-restraint stress (Shalini and Kumar, 2011). Additionally, the ethanolic extract demonstrated a significant dose-dependent anti-diarrheal activity by reducing the total number of faeces, weight, and volume of the intestinal contents (Al-Snafi, 2016; Al-Snafi, 2018). Additionally, these species have demonstrated numerous biological activities, including antidiabetic, cardioprotective, antihyperlipidemic, nephroprotective, cytotoxic, hepatoprotective, anti-inflammatory, antioxidant, antimicrobial, and anti-influenza A virus (IAV) activities (Zhang et al., 2010; Sriram, 2011; El-Tantawy and Saied, 2012; El-Tantawy et al., 2013b; Salem et al., 2013; Hossen et al., 2014; Kantheti et al., 2014; Pawar and Nasreen, 2018; Mamillapalli et al., 2020; Muthuraj et al., 2020; Abd Al Haleem et al., 2022; Aboulthana et al., 2022; Al-Hadad and AL-Zubaidy, 2022; Xu et al., 2022).

Last century, few species, including *Casuarina glauca*, *Casuarina cunninghamiana*, and C*. equisetifolia*, were introduced into Egypt where they were mostly planted as windbreaks and to provide wood for resident industries since *Casuarina* wood is characterized by its hardness, heaviness, and its dark red colour (El-Kader et al., 2021). One of the most extensively planted Casuarina species is *Casuarina glauca* Sieb. Ex Spreng., a medium sized deciduous tree with 8–20 m height, which is also known as swamp oak, river oak, or swamp-she oak (Zhong et al., 2013).

To our knowledge, no previous investigations have been conducted on the phytochemical composition of *C. glauca* branchlets extract nor its probable biological activities, specifically in regard to its effectiveness as a remedy for colitis. Therefore, determining the chemical profile of *C. glauca* branchlets ethanolic extract (CGBRE) and investigating its potential efficacy as a treatment for UC utilising *in silico* approach followed by *in vivo* validation experiments were the objectives of the current study.

## 2 Materials and methods

### 2.1 Plant material and extraction

*C. glauca* Sieb. ex Spreng. fresh branchlets were collected in April 2022 from the Experimental Farm of El-Kassasin Horticultural Research Station, Horticulture Research Institute, Agriculture Research Center (ARC), Giza, Egypt. The plant was taxonomically verified by Eng. Therese Labib Youssef, a Plant Taxonomy Consultant at the Ministry of Agriculture and Ex Manager at El-Orman Botanical Garden, Giza; and Prof. Ahmed Abd El Dayem, Professor at Forestry Department, Horticulture Research Institute, Giza, Egypt. The Department of Pharmacognosy at the Faculty of Pharmacy, Zagazig University, has preserved a specimen voucher in their Herbarium. The voucher has been assigned a unique Registration Number, ZU-Ph-Cog-0303, for identification purposes.

The shade dried powdered branchlets (300 g) was extracted by maceration with 70% ethyl alcohol (3 × 1 L). The extract was subjected to low-pressure evaporation to provide a viscous residue of 60 g weight.

### 2.2 LC-ESI-MS/MS metabolite profiling of CGBRE

A precise amount of CGBRE (50 mg) was dissolved in a solution comprising water:methanol:acetonitrile (50:25:25) with a volume of 1 mL. The resultant mixture was subjected to vortexing for 2 min, followed by ultrasonication for 10 min, and subsequently centrifuged for 10 min at 1,000 rpm. In accordance with the methodology previously outlined by Mohamed et al. (2022), a 50 µL sample solution was subjected to dilution with the mobile phase to a volume of 1,000 µL. Subsequently, 10 L of the resulting solution, with a concentration of 2.5 μg/μL, was analyzed via LC-ESI-MS/MS in negative mode, utilizing the ExionLCTM AD UPLC instrument and a TripleTOF 5600+Time-of-Flight Tandem Mass Spectrometer (AB SCIEX). The present study employed in-line filter disks (Phenomenex^®^, Torrance, United States) measuring 0.5 μm × 3.0 mm as a pre-column, and X select HSS T3 (Waters^®^, Milford, MA, United States) measuring 2.5 μm and 2.1 mm × 150 mm, operating at 40°C, as the analytical column. The experimental conditions for the chromatographic analysis involved setting the column temperature and flow rate at 40 0C and 0.3 mL/min, respectively. The mobile phases utilized in the analysis were composed of two buffers, namely, buffer A and buffer B. Buffer A was comprised of 5 mM ammonium format buffer pH 8% and 1% methanol, while buffer B was composed of 100% acetonitrile. The elution process was initiated with a ratio of 90 (A): 10 (B) for the first 20 min, followed by a transition to a ratio of 10 (A): 90 (B) from 21 to 25 min, and finally a return to the initial ratio for the last 3 min until the conclusion of the analysis at 28 min. In order to discern chemical compounds, the PeakViewTM software was employed to conduct a comparative analysis of retention time (RT) and mass-to-charge ratio (m/z) values obtained through MS and MS/MS. The XIC Manager feature within the PeakViewTM software was utilized to ascertain peak area values. Extracted ion chromatograms (XICs) were automatically generated for each compound and subsequently compared to a threshold value defined by the user.

### 2.3 Network analysis (network pharmacology)

#### 2.3.1 Obtaining pharmacokinetics of CGBRE major compounds and their related targets

To obtain pharmacokinetics of CGBRE major constituents, first their SMILES were attained from PubChem (https://pubchem.ncbi.nlm.nih.gov/, accessed on 1–3 March 2023) database or using ChemDraw v20.0.0.41 (PerkinElmer Informatics, Inc., United Kingdom). Then, the pharmacokinetics parameters of the major compounds were obtained from the SwissADME web tool (http://www.swissadme.ch/, accessed on 4–5 March 2023) (Daina et al., 2019).

To obtain the molecular targets associated with CGBRE major compounds, the SwissTargetPrediction (http://www.swisstargetprediction.ch/, accessed on 6–7 March 2023) database was used (Daina et al., 2019).

#### 2.3.2 Identification of targets associated with UC

To identify the molecular targets/proteins linked to UC, three databases were used; DisGeNeT (https://www.disgenet.org/search, accessed on 8 March 2023) (Piñero et al., 2020), MalaCards (https://www.malacards.org/, accessed on 8 March 2023) (Rebhan et al., 1997; Safran et al., 2010), and Online Mendelian Inheritance in Man (OMIM, https://www.omim.org/, accessed on 8 March 2023) (Hamosh et al., 2005).

#### 2.3.3 Disease-extract overlapping targets and construction of protein–protein interactions and compound-target networks

To identify the intersecting protein targets between major constituents of CGBRE and UC, Microsoft Excel was utilized, and the overlap was shown as a Venn diagram.

To construct the protein–protein interactions (PPI) network of the intersected protein targets, experimentally validated PPIs were included from two databases; the STRING database Version 11.5 (https://string-db.org/, accessed on 9 March 2023) (Szklarczyk et al., 2021) and the IID database Version 2021–05 (http://iid.ophid.utoronto.ca/, accessed on 9 March 2023) (Kotlyar et al., 2022).

After construction of the PPI network, a compound-target (compound-protein) network was also built; this network contained the interactions of the major compounds of CGBRE with the overlapping target proteins. To visualize the networks, the Cytoscape 3.10.1 program (NIGMS, United States) (Shannon et al., 2003) was utilized. Ranking of the protein targets was performed based on degree and betweenness centrality measures, while the compounds of CGBRE were ranked based on the degree value through the implementation of the CytoHubba plugin in Cytoscape (Chin et al., 2014).

#### 2.3.4 The enrichment analysis

To perform the GO analysis and Kyoto Encyclopedia of Genes and Genomes (KEGG) pathway enrichment, g:Profiler enrichment tool was used (https://biit.cs.ut.ee/gprofiler/gost, accessed on 27 May 2023) (Raudvere et al., 2019). A *p*-value <0.05 was used as a cutoff. *Homo sapiens* (Human) was chosen as organism, and GO molecular function, GO cellular component, GO biological process and KEGG were selected as data sources.

### 2.4 Molecular docking study

The present study employed the Molecular Operating Environment MOE version 2019.0102 software (Chemical Computing Group, Montreal, CA) for conducting molecular docking investigations. The crystal structures of epidermal growth factor receptor tyrosine kinase (EGFR; PDB: 1M17) (Stamos et al., 2002), leucine-rich repeat serine/threonine-protein kinase 2 (LRRK2; PDB: 6DLO) (Zhang et al., 2019b), and heat shock protein HSP 90-Alpha in complex with T5M (HSP90AA1; PDB: 2XHX) (Murray et al., 2010) were sourced from the Protein Data Bank (http://www.rcsb.org, accessed on 7 March 2023) (Burley et al., 2022). The protein structures of EGFR, LRRK2, and HSP90AA1 were individually prepared using the MOE Quick Preparation tool with the Amber10: EHT forcefield. The chemical compounds, namely, caffeic acid, quercetin, tricin, quinic acid, kaempferol-3*-O-*rhamnoside, 3,11,17 trihydroxytricyclo [12.3.1.12,6]-nonadeca-1(18),2(19),3,5,14,16-hexaen-8-one, gallic acid, caffeic acid 4*-O-*glucoside, kaempferol-3*-O-*arabinoside, and coumaroyl quinic acid, were drawn using Chemdraw^®^ (PerkinElmer Informatics, Inc., United Kingdom) and subsequently transferred to MOE using smiles canonical. The energy of each component was minimized using a root mean square gradient of 0.1 kcal/mol/Å2. Subsequently, each component structure was protonated at pH 7.4, and a database file was prepared. To validate the active site, the co-crystallized ligands for both HSP90A and EGFR were re-docked. However, as LRRK2 lacked a co-crystallized ligand, the geometrical approach of MOE Site Finder was utilized for active pocket prediction. The exploited docking placement methodology is triangle matcher. Each ligand was allowed to be flexible while the protein structure was kept rigid. The scores of the docking energy for the best-fitted poses of the components with the protein active pocket were recorded.

### 2.5 *In Vivo* Experiments

#### 2.5.1 Animals, ethical statement and experimental design

The Institutional Animal Care and Use Committee (IACUC) at Zagazig University has granted approval for all experiments conducted, with a reference number ZU-IACUC/3/F/82/2023. The procedures followed were in strict adherence to the Animal Research: Reporting of *In Vivo* Experiments (ARRIVE) guidelines.

In this study, a total of 24 male Wistar rats weighing between 180–220 g were utilized. The animals were sourced from the animal facility at the Faculty of Veterinary Medicine, Zagazig University, Zagazig, Egypt. Throughout the study, the rats were provided with standard chow diet and water *ad libitum*. One week prior to the commencement of the experiments, the rats were acclimatized to standard conditions, including a temperature of 24°C, 50%–60% humidity, and a 12:12 h light/dark cycle. The rats were randomly assigned to one of three groups, including a control group (n = 8), an UC group (n = 8), and an UC group treated with CGBRE (n = 8). Animals were treated orally with 300 mg/kg CGBRE suspension or vehicle (1% gum acacia) for 6 days after colitis induction. Extract dose selection was based on a previous study of another *Casuarina* species (*C. equisetifolia*) tested in acute chemical-induced nephrotoxicity in rats (El-Tantawy et al., 2013a), beside some preliminary studies. In the later, a pilot study was performed to compare the effectiveness of different doses of CGBRE (n = 5). Results of the pilot study (Supplementary Figure S1) showed that 300 mg/kg was the most effective in alleviating colitis and thus was selected in the main study.

#### 2.5.2 Colitis induction and sample collection

Ulcerative colitis (UC) was induced in rats as previously described (Saber et al., 2019). Briefly, prior to the experimental procedure, rats were subjected to a 24-h fast. On the day of induction, rats were anaesthetized with ketamine/xylazine (50 and 10 mg/kg i. p, respectively). Acetic acid (3% v/v in saline, 2 mL) was dripped via 6F polypropylene catheter lubricated with Vaseline and inserted through the anal canal into the colon. Saline was used instead of acetic acid in the control group. After the treatment period, the animals were humanely euthanized to facilitate sample collection. To calculate the colon weight/length ratio, colons were carefully weighed, and their length was precisely measured. Thereafter, sections of the colons were preserved in 10% buffered formalin for subsequent histological and immunohistochemical analyses, while the remaining portions were rapidly frozen in liquid nitrogen and stored at a temperature of −80°C.

#### 2.5.3 Disease severity evaluation

To assess disease severity, a disease activity index (DAI) was used according to previous studies (Cooper et al., 1993; Saber et al., 2019; Fouad et al., 2021). Macroscopic inflammation score was evaluated according to previous studies (Jagtap et al., 2004; Saber et al., 2019). The DAI scoring system and macroscopic inflammation score are presented in Table 1.

**TABLE 1:** DAI scoring and macroscopic inflammation score.

	Parameter	Score				
		0	1	2	3	4
DAI	**% Body weight loss**	None	1%–5%	6%–10%	11%–20%	>20%
	**Diarrhea**	Normal	Loose stool		Watery diarrhea	
	**Bloody stool**	Normal	Slight bleeding		Gross bleeding	
Macroscopic inflammation	**Macroscopic changes**	No changes	Mucosal erythema	Mild mucosal edema, slight bleeding or small erosions	Moderate edema, slight bleeding ulcers or erosions	Severe ulceration, edema and tissue necrosis

#### 2.5.4 Histopathology

The colon tissues were subjected to fixation in 10% neutral buffered formalin for a period of 72 h. Subsequently, they were processed through a series of ethanol grades, cleared in Xylene, and infiltrated and embedded in Paraplast tissue embedding media. Following this, 5 µm thick serial sections were obtained using a rotatory microtome and mounted on glass slides. The tissue sections were subjected to staining using Hematoxylin and Eosin, which is a standard staining method employed for blinded light microscopic examination. The examination was conducted by an experienced histologist.

#### 2.5.5 Immunohistochemistry

Paraffin-embedded sections measuring 5 µm were subjected to deparaffinization and treated with 0.3% H_2_O_2_ for 20 min. The sections were then incubated overnight at 4°C with anti-EGFR “sc-373746”, anti-HSP90 “sc-515081”, anti-NFkB p65 “sc-8008” (Santa Cruz Biotechnology, Inc., CA, USA-1:100), or anti-cleaved Caspase-3 “GB11532” (Service bio. Wuhan, China-1: 300). Following this, the slides were rinsed with PBS and incubated with the secondary antibody HRP Envision kit (DAKO) for 30 min at room temperature. The slides were then rinsed and incubated with DAB for 15 min. Subsequently, they were rinsed with PBS, counterstained with Mayer’s hematoxylin, dehydrated, and cleared in xylene. Finally, the slides were cover-slipped for microscopic examination. Morphometric analysis for immunohistochemistry was performed using the image analysis software, ImageJ2 v2.14 (NIH, United States).

#### 2.5.6 ELISA

The Leucine-rich repeat serine threonine-protein kinase 2 (LRRK2) was quantified in colon tissue homogenate utilizing the Rat LRRK2 ELISA Kit (E3407Ra, Bioassay Technology Laboratory BT LAB, Shanghai, China) in accordance with the manufacturer’s instructions. Quantification was based on the obtained standard curve using Four parameter logistic (4 PL) regression model.

#### 2.5.7 Statistical analysis

The statistical analyses were conducted using GraphPad Prism version 10.0.0 (CA, United States). The results were reported as means ± standard deviation (SD). The animal groups were compared using the one-way analysis of variance (ANOVA) and applying the posthoc Tukey’s multiple comparison test. Statistical significance was considered at *p* < 0.05.

## 3 Results

### 3.1 LC-ESI-MS/MS analysis of CGBRE

**FIGURE 1:**
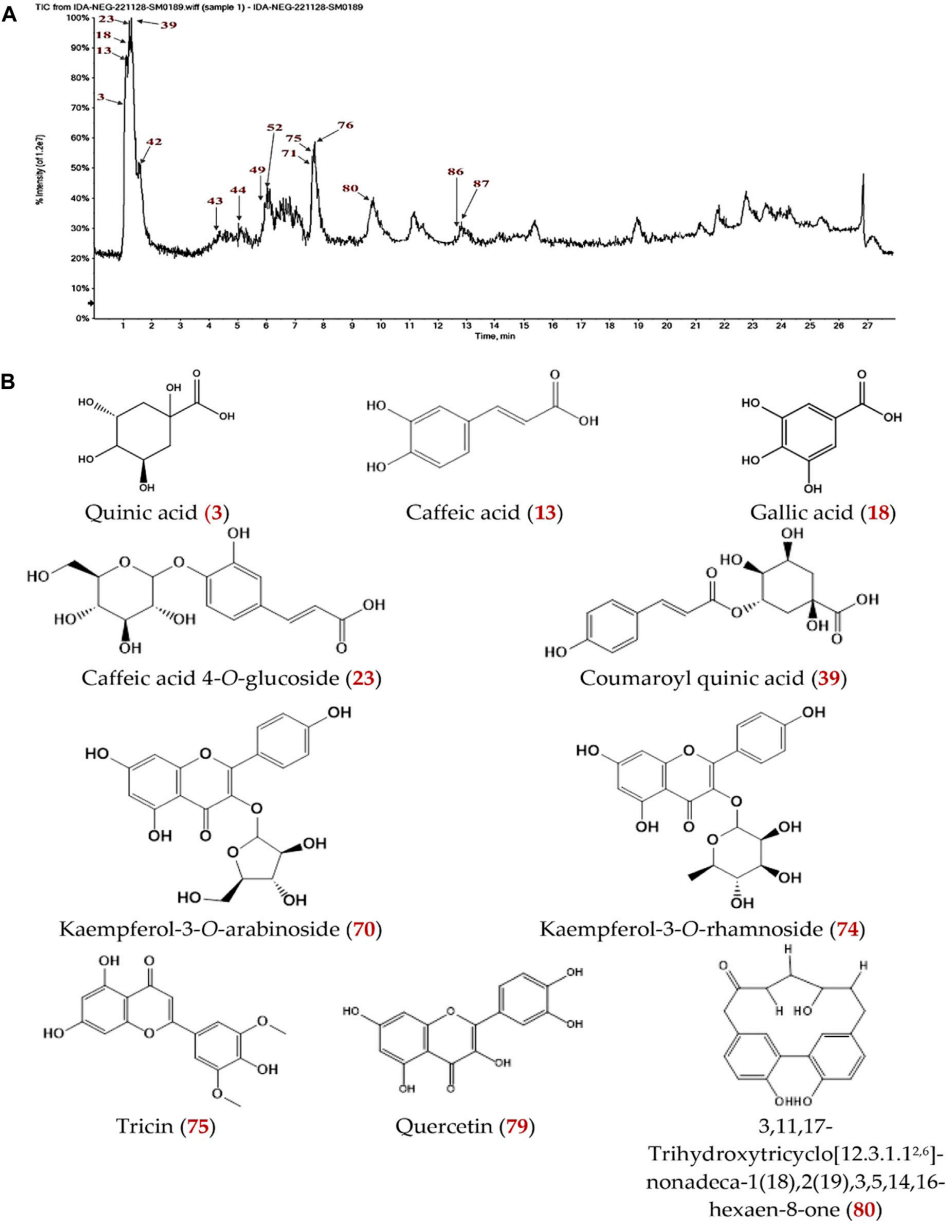
**(A)** LC-MS total ion chromatogram of CGBRE in negative ion mode. **(B)** The structure of some LC-MS identified major components of CGBRE. Numbers in red (in a and b) are related to Table 2.

The secondary metabolites of CGBRE were identified in negative mode through liquid chromatography with mass spectrometry (LC-ESI-MS/MS) analysis. Table 2 presents the presence of 86 compounds belonging to various phytochemical classes, including organic and phenolic acids, their derivatives, flavonoid aglycones and glycosides, cyclic and non-cyclic diarylheptanoid and their glycosides, polyphenols, sugars, sugar acid, anthraquinone, and lignan glucoside. The identification of these compounds was tentatively done by comparing their retention times and mass spectrometric fragmentations with data from the literature. The total ion chromatogram (TIC) of CGBRE in negative mode is illustrated in Figure 1.

**TABLE 2:** Phytochemical profiling of CGBRE by LC-ESI-MS/MS in negative mode.

No.	Rt	[M-H]^-^	MS^2^ fragments (m/z)	Tentative identification	Class	Ref
1	0.985	133	115, 89, 72, 71, 59	Malic acid	Organic acid	Aboulthana et al. (2022)
2	0.986	187	143, 125	Hydroxybenzoic acid dv	Phenolic acid dv	Ağalar et al. (2018)
3	1.000	191.019	173, 171, 127, 111, 93, 87	Quinic acid	Cyclohexanecarboxylic acid	Aboulthana et al. (2022)
4	1.002	289.017	245	(Epi) catechin	Flavanol (procyanidin monomer)	Mohamed et al. (2022)
5	1.013	224.905	181, 119	Dihydrosinapic acid	Phenolic acid	Tang et al. (2019)
6	1.015	300.953	283, 255	Pentahydroxyflavone (Viscidulin I)	Favonol	Chang et al. (2016)
7	1.016	344.910	301, 283	Rosmanol	polyphenol	Christaki et al. (2022)
8	1.017	405.101	191	Quinic acid derivative	Cyclohexanecarboxylic acid dv	Tentatively identified
9	1.027	259.043	191	Quinic acid derivative	Cyclohexanecarboxylic acid dv	Tentatively identified
10	1.041	297.020	253, 235	2-carboxy-3,8-dihydroxy-1-methylanthraquinone	Anthraquinone	Zhao et al. (2013)
11	1.075	135.029	117, 108, 89, 75	L-Threonic acid	Sugar acid	Cartabia et al. (2021)
12	1.076	173.047	155, 137, 129, 111, 93	Shikimic acid	Cyclohexanecarboxylic acid	Mohamed et al. (2022)
13	1.076	179.056	161, 143, 135, 99, 75	Caffeic acid	Phenolic acid	Mohamed et al. (2022)
14	1.079	331.067	169, 125	Monogalloyl glucose	Phenolic acid glucoside	Attia et al. (2022)
15	1.092	457.045	265, 191	Quinic acid derivative	Cyclohexanecarboxylic acid dv	Tentatively identified
16	1.101	125.025	107	1,3,5-Trihydroxybenzene	polyphenol	Ağalar et al. (2018)
17	1.102	164.927	147, 121, 105, 99, 87	3-(3-Hydroxyphenyl)propionic acid or 3-(4-hydroxyphenyl)propionic acid	Phenolic acid	López-Fernández et al. (2020)
18	1.102	169.015	125	Gallic acid	Phenolic acid	Mohamed et al. (2022)
19	1.103	179.056	161, 143, 75	Hexose	Monosaccharide	Verardo et al. (2009)
20	1.152	153.019	109	Dihydroxybenzoic acid (protocatechuic acid)	Phenolic acid	Mohamed et al. (2022)
21	1.163	179.056	161, 143	Hexose	Monosaccharide	Verardo et al. (2009)
22	1.165	377.085	341, 215, 179, 119, 89, 59	Disaccharides chloride adduct	Disaccharide	Zhan et al. (2021)
23	1.176	341.109	179, 161, 135	Caffeic acid 4-*O*-glucoside	Phenolic acid glucoside	Karar et al. (2016)
24	1.178	401.127	341, 191, 179, 161, 135	Caffeic acid hexoside derivative	Phenolic acid hexoside dv	Tentatively identified
25	1.188	329.087	167	Hydroxy methoxy benzoic acid hexoside (vanillic acid glucoside)	Phenolic acid glucoside	Al-Yousef et al. (2020)
26	1.202	315.070	153	Protocatechuic acid 4-glucoside	Phenolic acid glucoside	Mohamed et al. (2022)
27	1.228	341.109	179, 161, 131, 119	Sucrose	Disaccharide	Saeed et al. (2022)
28	1.239	187.098	169, 125	Hydroxygallic acid	Phenolic acid	Fathoni et al. (2017)
29	1.241	291.013	247, 175	Brevifolin carboxylic acid	Isochromene-1-carboxylic acid (phenolic acid)	Mekam et al. (2019)
30	1.244	533.172	191	Quinic acid derivative	Cyclohexanecarboxylic acid	Spínola et al. (2015)
31	1.245	683.227	341, 221, 179	Hexose polymer	Polysaccharide	Llorent-Martínez et al. (2015)
32	1.269	477.083	409, 341, 179, 161, 135	Calceolarioside A	Phenolic acid	Li et al. (2015b)
33	1.270	549.168	503, 221, 179	Hexose polymer	Polysaccharide	Llorent-Martínez et al. (2015)
34	1.283	683.227	341, 179	Caffeic acid hexoside dimer	Phenolic acid	Silva et al. (2019)
35	1.291	341.109	179, 161, 143, 131, 119	Trehalose	Disaccharide	Saeed et al. (2022)
36	1.295	477.083	409, 341, 179, 161, 135	Calceolarioside B	Phenolic acid	Li et al. (2015b)
37	1.302	163.040	119	Coumaric acid	Phenolic acid	El-sayed et al. (2021)
38	1.315	146.960	129, 103	Cinnamic acid	Phenolic acid	El-Shazly et al. (2021)
39	1.317	337.094	191, 173, 163, 119	Coumaroyl quinic acid	Phenolic acid	Liu et al. (2022)
40	1.320	521.173	341, 191, 179, 161, 119	Hexose polymer	Polysaccharide	Silva (2019)
41	1.356	341.108	179, 161, 143, 131, 119	Maltose	Disaccharide	Saeed et al. (2022)
42	1.431	166.957	152, 123	Methoxy hydroxy benzoic acid (Vanillic acid)	Phenolic acid	Mohamed et al. (2022)
43	4.519	447.056	315, 314, 300, 299, 271	Isorhamnetin-*O*-pentoside	Flavonol glycoside	Agarwal et al. (2021)
44	5.304	593.152	447, 431, 285	Kaempferol-*O*-hexoside-*O*-rhamnoside	Flavonol glycoside	Llorent-Martínez et al. (2016)
45	5.441	449.075	317, 316	Myricetin-3-*O*-pentoside	Flavonol glycoside	Hadjadj et al. (2020)
46	5.733	609.147	301, 300	Quercetin-3-*O*-rutinoside	Flavonol glycoside	Borges et al. (2010)
47	5.858	491.191	329, 287	Cyanidin-3-*O*-acetylglucoside	Anthocyanin	El-sayed et al. (2021)
48	5.939	463.087	301, 300, 271, 255	Quercetin-3-*O*-galactoside	Flavonol glycoside	Borges et al. (2010)
49	5.941	523.214	361	Secoisolariciresinol-9-*O*-glucoside	Lignan glycoside	Xiao et al. (2018)
50	5.942	599.073	463, 301	Quercetin-3-*O*- hexoside- protocatechoic acid	Flavonol glycoside dv	Osman et al. (2021)
51	6.043	463.089	301, 300, 271, 255	Quercetin-3-*O*-glucoside	Flavonol glycoside	Borges et al. (2010)
52	6.089	435.139	345, 315, 273	Phloretin-*C*-glucoside (nothofagin)	Dihydrochalcone glucoside	Jiménez Sánchez (2016)
53	6.092	497.178	482, 335, 313, 169	Methyl Di-O-Galloylglucopyranoside	Phenolic acid glycoside	Li et al. (2015a)
54	6.103	181.051	166, 151	Syringaldehyde	Phenolic aldhyde	Sanz et al. (2012)
55	6.131	615.099	463, 407, 301	Quercetin-*O*-galloyl-hexoside	Flavonol glycoside	Li and Seeram (2018)
56	6.203	475.155	295	Platyphylloside (Platyphyllonol-hexoside)	linear diarylheptanoid glycoside	Alberti et al. (2018)
57	6.290	633.070	463, 301, 275	Galloyl-HHDP-glucoside (corilagin)	Ellagitannin	Samet et al. (2022)
58	6.343	433.076	301, 271, 255	Quercetin 3-*O*-xyloside	Flavonol glycoside	Olennikov et al. (2020)
59	6.467	449.100	287	Dihydrokaempferol glucoside	Flavanonol glycoside	Razgonova et al. (2021)
60	6.468	473.180	311, 179	Caffeoyl hexosyl pentoside	Phenolic acid glycoside	Tentatively identified
61	6.479	433.75	301, 271, 255	Quercetin 3-*O*-arabinoside	Flavonol glycoside	Olennikov et al. (2020)
62	6.480	447.093	285, 284, 255, 227	kaempferol-3-*O*-galactoside	Flavonol glycoside	Borges et al. (2010)
63	6.484	593.149	285, 284	Kaempferol-*O*-rutinoside	Flavonol glycoside	Maisto et al. (2022)
64	6.510	447.091	285, 284, 255, 227	kaempferol-3-*O*-glucoside	Flavonol glycoside	Borges et al. (2010)
65	6.670	441.079	289, 169	Catechin-*O*-galloyl	Procyanidin	Zhao et al. (2013)
66	6.707	327.125	211, 197, 193	Casuarinondiol	Cyclic diarylheptanoid	Felegyi-Tóth et al. (2022)
67	6.834	477.103	315, 314	Isorhamnetin-3-*O*-glucoside	Flavonol glycoside	Mohamed et al. (2022)
68	6.872	447.092	301,300	Quercetin-3-*O*-rhamnoside	Flavonol glycoside	Borges et al. (2010)
69	7.113	417.083	285, 284, 255, 227	Kaempferol 3-*O*-xyloside	Flavonol glycoside	Jelača et al. (2022)
70	7.139	417.082	285, 284, 255, 227	Kaempferol-3-*O*-arabinoside	Flavonol glycoside	Wang and Chen (2022)
71	7.241	445.136	295, 189	Platyphyllonol-pentoside	Linear diarylheptanoid glycoside	Alberti et al. (2018)
72	7.264	473.183	293, 89	5-Hydroxy-1,7-bis-(4-hydroxyphenyl)-hept-4-en-3-one-hexoside	Linear diarylheptanoid glycoside	Alberti et al. (2018)
73	7.439	435.130	273, 167	Phloretin-2-*O*-glucoside (phloridzin)	Dihydrochalcone glycoside	Sánchez-Rabaneda et al. (2004)
74	7.597	431.094	285, 284, 255, 227	Kaempferol-3-*O*-rhamnoside	Flavonol glycoside	Abdelaziz et al. (2020)
75	7.661	329.234	229, 211, 183	3′,5′-*O*-dimethyltricetin (Tricin)	Flavone	Ağalar et al. (2018)
76	8.350	609.128	463, 447, 301, 300	Quercetin-*O*-hexoside-*O*-rhamnoside	Flavonol glycoside	Llorent-Martínez et al. (2016)
77	8.590	459.314	415, 297	Afromosin 7-*O*-*β*-D-glucopyranoside	Isoflavone glycoside	Tchoumtchoua et al. (2013)
78	9.010	435.109	289	Catechin-7-*O*-rhamnoside	Flavanol glycoside	Reed (2009)
79	9.198	301.035	179,151	Quercetin	Flavonol	Mohamed et al. (2022)
80	9.294	311.129	267, 253	3,11,17-trihydroxytricyclo [12.3.1.1^2,6^]-nonadeca-1(18),2(19),3,5,14,16-hexaen-8-one	Cyclic diarylheptanoid	Felegyi-Tóth et al. (2022)
81	9.666	579.115	433, 301, 300	Quercetin-3-*O*-pentoside–7-*O*-deoxyhexoside	Flavonol glycoside	Jelača et al. (2022)
82	9.826	313.145	207, 189, 163, 149	5-hydroxy-1,7-bis-(4′-hydroxyphenyl)-3-heptanone (5-hydroxy-3-platyphyllone) (Platyphyllonol)	linear diarylheptanoid	Felegyi-Tóth et al. (2022)
83	10.354	593.130	447, 301,300	Quercetin-3-*O*-rhamnoside-7-*O*-rhamnoside	Flavonol glycoside	Onkokesung et al. (2014)
84	10.629	285.041	257, 229, 151	kaempferol	Flavonol	Fathoni et al. (2017)
85	12.708	293.118	83	Alnusone	Cyclic diarylheptanoid	Alberti et al. (2018)
86	12.780	587.239	293	Giffonin I	Cyclic diarylheptanoid glycoside	Alberti et al. (2018)

#### 3.1.1 Characterization of flavonoid aglycones

Identification of different flavonoids aglycons revealed the presence of flavanol (catechin **4**), Flavonol (Viscidulin I **6**), flavonols (quercetin **79** and kaempferol **84**) and flavone (tricin **75**).

#### 3.1.2 Characterization of flavonoid glycosides

Most of the recognized flavonoid glycosides were found to be flavonol glycosides as kaempferol, quercetin, isorhamnetin and myricetin derivatives. Compounds **44**, **62–64**, **69**, **70** and **74** shared the common ion fragment at m/z 285 corresponding for the aglycone kaempferol. Similarly, the common ion fragment at m/z 301 (quercetin) was observed in MS/MS fragmentations of compounds **46**, **48**, **50**, **51**, **55**, **58**, **61**, **68**, **78**, **81 and 83**. Compounds **42** and **67** are characterized by the presence of ion fragments at m/z 315 for the aglycone moiety isorhamnetin. The ion fragment at 317 (for myricetin) was detected for compound **45**. Additionally, both **65** and **78** displayed the appearance of typical fragment ion at m/z 289 (catechin) which recognized for flavanol derivatives (catechin*-O-*galloyl and catechin-7*-O-*rhamnoside, respectively), besides recognition of minor flavanonol glycoside as dihydrokaempferol glucoside (**59**), dihydrochalcone glycoside as phloretin-2*-O-*glucoside (**73**) and isoflavone glycoside as afromosin 7*-O-β*-D-glucopyranoside (**77**).

#### 3.1.3 Characterization of phenolic acids, carboxylic and/or their derivatives

Most of the identified phenolic acids are caffeic, 3-(3-Hydroxyphenyl) propionic, gallic, dihydrosinapic, vanillic, protocatechuic, brevifolin carboxylic, calceolarioside A, coumaric, cinnamic acids and/or their glycosides. Additionally, some carboxylic acids were detected as malic, quinic, L-threonic and shikimic acids.

#### 3.1.4 Characterization of diarylheptanoids

Diarylheptanoids are a class of phenolic compounds with the aryl-C_7_-aryl structure, they were identified in the extract either aglycones or glycosides. Four linear (compounds **56**, **71**, **72** and **82**) and four cyclic (compounds **66**, **80**, **85** and **86**) diarylheptanoids were established via the reported mass spectrometry data and characteristic fragmentation pattern. Regarding linear diarylheptanoids, compound **82**and its glycosides **(**compounds **56** and **71**) produced a deprotonated molecular ion peak at *m/z* 313.145, 475.155 and 445.136, respectively. Compounds **56** and **71** shared the common ion fragment at *m/z* 295 corresponding for the aglycone (platyphyllonol-18 Da), due to neutral loss of hexose moiety (180 Da) and pentose moiety (150 Da), respectively, confirming that both are glycosylated through the OH group on C_7_ chain (Alberti et al., 2018). Compound **82** displayed its fragment ions at *m/z* 207, 163, 149, hence identified to be platyphyllonol (Felegyi-Tóth et al., 2022). Similarly, compound **72** was fragmented to give ion at *m/z* 293 due to neutral loss of hexose moiety (180 Da). In relation to cyclic compounds, compound **66** generated an [M-H]^-^ ion at m/z 327.125 and a representative fragment ion at m/z 211, which was attributed to the neutral loss of hydroxylated oxopentanal. This loss occurred through a rearrangement of the pseudomolecular ion, followed by the cleavage of two C-C bonds (C_7_-C_8_ and C_12_-C_13_).

This fragmentation pattern is indicative of cyclic diarylheptanoids possessing a meta, meta-cyclophane structure. Therefore, compound **66** was identified as casuarinondiol. In a similar way, compound **80** showed [M-H]^-^ at *m*/*z* 311.129, fragments ion at *m/z* 211 and 267. The latter is due to the neutral loss of an ethenol unit through the breakdown of two C- C bonds (C_7_-C_8_ and C_9_-C_10_), thus The compound **80** was identified as 3,11,17-trihydroxytricyclo [12.3.1.1^2,6^]- nonadeca-1(18),2(19),3,5,14,16-hexaen-8-one. Compound **85** and its glycosides, compound **86**, displayed [M-H]^-^ at *m*/*z* 293.118 and 587.239, respectively. Alnusone (**85**) exhibited simple fragmentation pathway led to formation of fragment ion at *m*/*z* 83 due to neutral loss of 210 Da (diaryl moiety) (Masullo et al., 2016). while MS^2^ fragmentation of compound **87** gave a characteristic fragment for the aglycone at *m*/*z* 293 resulting from neutral loss of hexosyl (162) and pentosyl (132), confirming that these sugar residues are linked to OH group of the aromatic rings (Alberti et al., 2018).

#### 3.1.5 Characterization of other compounds

Other compounds were recognized as polyphenols (rosmanol and 1,3,5-Trihydroxybenzene), anthraquinone (2-carboxy-3,8-dihydroxy-1-methyl anthraquinone), saccharides, Lignan glycoside (secoisolariciresinol-9*-O-*glucoside) and phenolic aldehyde (syringaldehyde).

### 3.2 The network pharmacology analysis

#### 3.2.1 Identification of CGBRE bioactive compounds

To recognize the possible bioactive constituents, the 38 major compounds of CGBRE, their pharmacokinetics and drug-likeness properties were screened (Supplementary Table S1). Of all compounds, 21 had no more than one violation of the Lipinski’s rule criteria. Therefore, these 21 compounds (Supplementary Table S2) were included for further analyses.

#### 3.2.2 The overlapping molecular targets of CGBRE bioactive compounds and UC

In order to ascertain the shared molecular targets between CGBRE bioactive compounds and UC, an initial step involved the acquisition of targets linked to the bioactive constituents of CGBRE was performed through the utilization of the SwissTargetPrediction database. Following the elimination of any duplicate entries, a total of 664 targets were identified (Supplementary Table S3).

**FIGURE 2:**
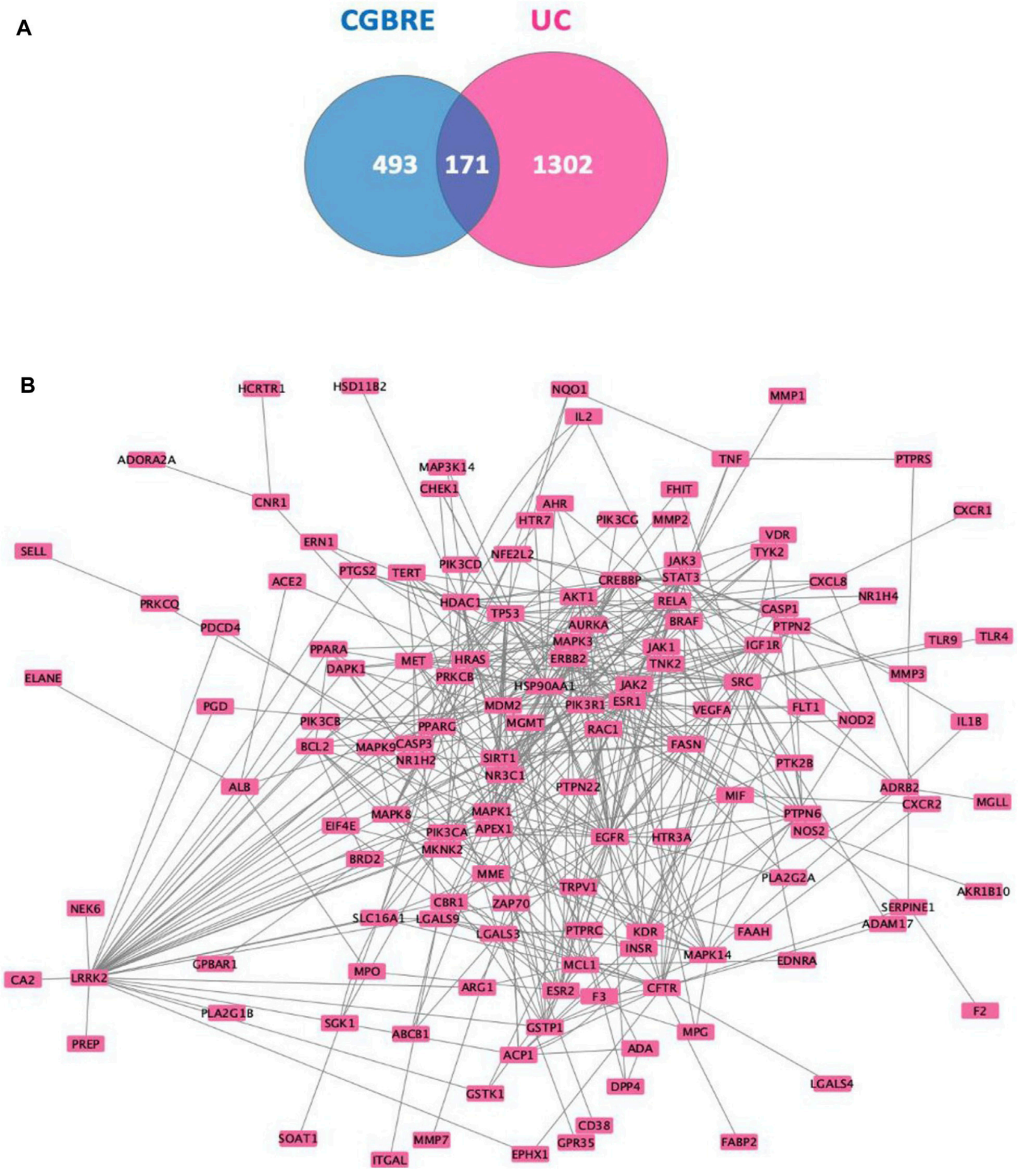
Common targets between CGBRE and UC and their PPI network. **(A)**. Venn diagram, **(B)**. PPI network. Abbreviations: CGBRE, *Casuarina glauca* branchlets ethanolic extract; PPI, protein-protein interactions; UC, ulcerative colitis.

Subsequently, the molecular targets related to UC were identified by utilizing three databases that are associated with disease targets, namely, DisGeNeT, MalaCards, and OMIM. A total of 1,586 targets were yielded, which were subsequently reduced to 1,473 after eliminating duplicates (Supplementary Table S4). Among the 664 CGBRE targets and the 1473 UC targets, 171 were found common (Supplementary Table S5, Figure 2A).

#### 3.2.3 Top CGBRE compounds targeting UC

To determine the CGBRE compounds that are associated with UC and their potential mechanism of action, initially, an experimentally validated protein-protein interactions (PPI) network of the common 171 targets was constructed using STRING and IID databases (Figure 2B). Then, the most relevant targets in the network were ranked based on their degree value, i.e., the number edges connected to each node. The uppermost targets included EGFR, LRRK2 and HSP90AA1 (degrees = 37, 36 and 34, respectively). Targets were also ranked based on betweenness centrality, and the top 20 targets of the degree-based ranking were the same as the top 20 targets, yet with changes in the rank value. Notably, the three top targets in the degree-based ranking, i.e., EGFR, HSP90 and LRRK2, were also found in the top ten targets of the betweenness-based ranking (rank 3, 6 and 10, respectively). Other targets that were ranked the fourth and fifth based on the degree, i.e., P53 and ESR1 became the two top targets based on betweenness (rank 2 and 1, respectively). Table 3 lists the top 20 targets with their relevant degree value.

**TABLE 3:** Top common targets ranked by Degree method.

Rank	Target name	Score
1	EGFR	37
2	LRRK2	36
3	HSP90AA1	34
4	TP53	28
4	ESR1	28
6	SRC	26
7	PIK3R1	22
7	STAT3	22
9	CFTR	20
10	MAPK1	19
10	HRAS	19
10	RELA	19
13	MDM2	18
14	CREBBP	17
14	AKT1	17
16	MAPK3	16
17	ESR2	15
17	MAPK8	15
17	HDAC1	15
20	LGALS3	14

**FIGURE 3:**
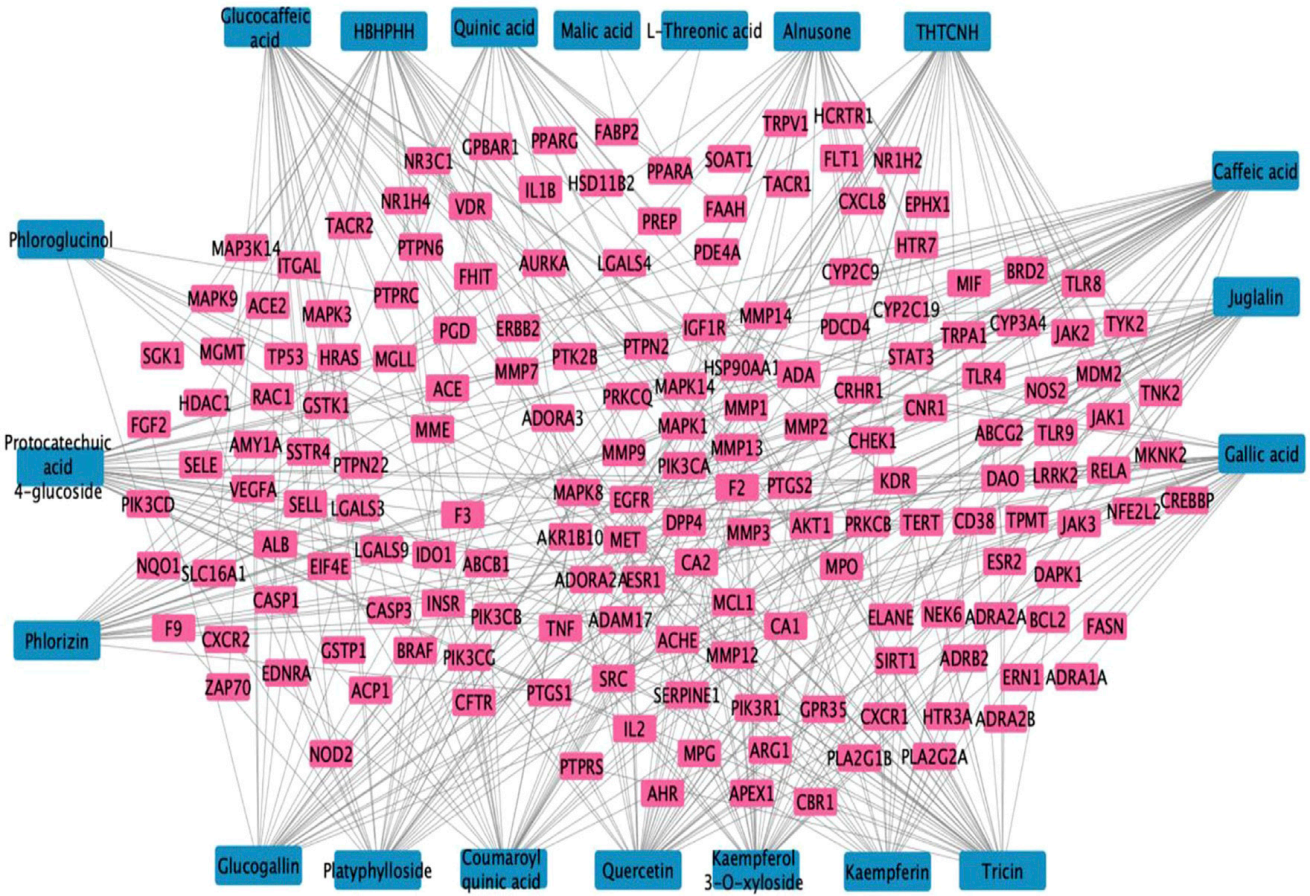
*Casuarina glauca* branchlets ethanolic extract (CGBRE) compounds-ulcerative colitis targets network.

After constructing the PPI network and identifying the relevant targets, the construction of a compound-target network was proceeded in Cytoscape (Figure 3) showing the association of the CGBRE bioactive compounds and the UC targets. The bioactive compounds were ranked based on degree value (Table 4), and the top compounds included caffeic acid, quercetin and tricin. Casuarinondiol did not have relevant targets on SwissTargetPrediction database.

**TABLE 4:** Bioactive compounds of CGBRE ranked by Degree method.

Rank	Compound	Score
1	Caffeic acid	40
2	Quercetin	36
3	Tricin	35
4	THTCNH (3,11,17-trihydroxytricyclo [12.3.1.1^2,6^]-nonadeca-1(18),2(19),3,5,14,16-hexaen-8-one)	32
5	Gallic acid	31
5	Coumaroyl quinic acid	31
7	HBHPHH (5-Hydroxy-1,7-bis-(4-hydroxyphenyl)-hept-4-en-3-one-hexoside)	30
7	Juglalin	30
7	Kaempferol 3-O-xyloside	30
10	Glucocaffeic acid	28
11	Platyphylloside	27
11	Alnusone	27
13	Glucogallin	26
13	Protocatechuic acid 4-glucoside	26
15	Quinic acid	23
15	Phlorizin	23
17	Kaempferin	21
18	Phloroglucinol	8
19	L-Threonic acid	2
19	Malic acid	2

#### 3.2.4 Enrichment analysis of the joint proteins

**FIGURE 4:**
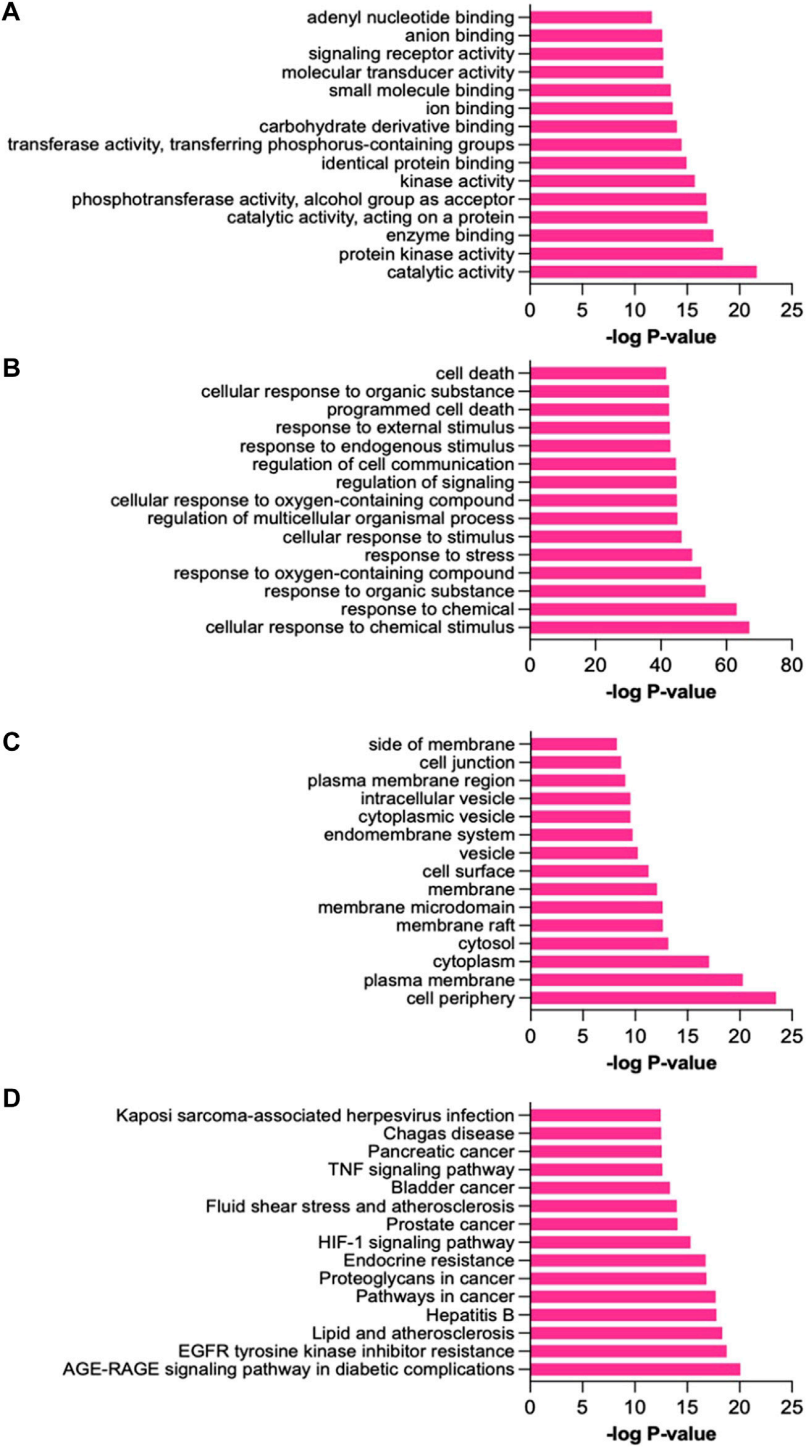
Enrichment analysis. **(A)**. Molecular functions, **(B)**. Biological processes, **(C)**. Cellular components, **(D)**. KEGG pathways.

To verify the significant characteristics of the 171 disease-compound joint proteins on a biological and functional level, Gene Ontology (GO) enrichment analysis was performed in molecular functions (MF), biological processes (BP), and cellular components (CC) (Figure 4A-c). Key MF included catalytic activity, enzyme binding and identical protein binding; key BP included cellular response to chemical stimulus, response to chemical and response to stress; key CC included cell periphery, plasma membrane and cytoplasm.

On biological process level, the top targets, e.g., EGFR, LRRK2, HSP90 and caspase proteins were enriched in cellular response to chemical stimulus, response to organic substance, response to oxygen-containing compound, response to stress, regulation of multicellular organismal process and many others. On molecular level, they were enriched in catalytic activity, protein kinase activity, enzyme binding and phosphotransferase activity among others. On cellular components level, they were in cell periphery, plasma membrane, cytoplasm, and many others.

To identify the potential pathways related to the actions of CGBRE in UC, KEGG pathway enrichment analysis of the 171 joint proteins was done (*p* < 0.05). Key enriched pathways encompassed AGE-RAGE signaling, EGFR tyrosine kinase and lipid and atherosclerosis pathways (Figure 4D). Details of GO and KEGG pathway analyses are displayed in Supplementary Table S6.

The enrichment analysis of the 171 UC targets showed that 26, 23 and 33 proteins are involved in AGE-RAGE, EGFR and atherosclerosis signaling pathways, respectively (Supplementary Table S6). These proteins included for instance BCL2, CREB, CXCL8, IL-1β, JAK2, MAPKs, STAT3, TLR4 and VEGFA among others. Indeed, previous studies on colitis showed that dysregulation of these proteins are associated with the pathogenesis of the disease (Broom et al., 2009; Coccia et al., 2012; Han and Theiss, 2014; Zhou et al., 2014; Hedl et al., 2016; Mateescu et al., 2017; Zhang et al., 2019; Shi et al., 2019; Zhu et al., 2021; Shahid et al., 2022).

Regarding the top targets in the network analysis, e.g., EGFR, LRRK2, HSP90 and caspase, these targets were found to be enriched in many pathways (Supplementary Table S6). For example, EGFR was found enriched in EGFR tyrosine kinase, cancer, PI3K-Akt signaling, relaxin signaling, foxO signaling, Rap1 signaling, MAPK signaling, JAK-STAT and calcium signaling pathways. LRRK2 was not enriched in any of the pathways. HSP90 was enriched in atherosclerosis, cancer, PI3K-Akt signaling, Th17 cell differentiation, IL-17 signaling, NOD-like receptor signaling and necroptosis. Caspase-3 was enriched in AGE-RAGE signaling, atherosclerosis, cancer, TNF signaling, IL-17 signaling, MAPK signaling, apoptosis and natural killer cell mediated cytotoxicity.

### 3.3 Molecular docking study

The objective of the present investigation was to evaluate the binding interaction between CGBRE components and key molecular targets in UC through a molecular docking analysis. The analysis was conducted using ten compounds (Table 4) that were selected based on their peak area values and scores derived from the degree value (Table 4). The focus of the analysis was on the three top targets of UC, namely, EGFR, LRRK2, and HSP90.

To accomplish this objective, the crystal structures of epidermal growth factor receptor (EGFR) tyrosine kinase in complex with erlotinib (PDB: 1M17) (Stamos et al., 2002), leucine-rich repeat serine/threonine-protein kinase 2; LRRK2 (PDB: 6DLO) (Zhang et al., 2019b), and heat shock protein HSP 90-Alpha in complex with T5M (PDB: 2XHX) (Murray et al., 2010) were utilized. The study aimed to determine the binding affinity of CGBRE components with the active pockets of targeted proteins.

#### 3.3.1 Docking with epidermal growth factor receptor (EGFR)

**FIGURE 5:**
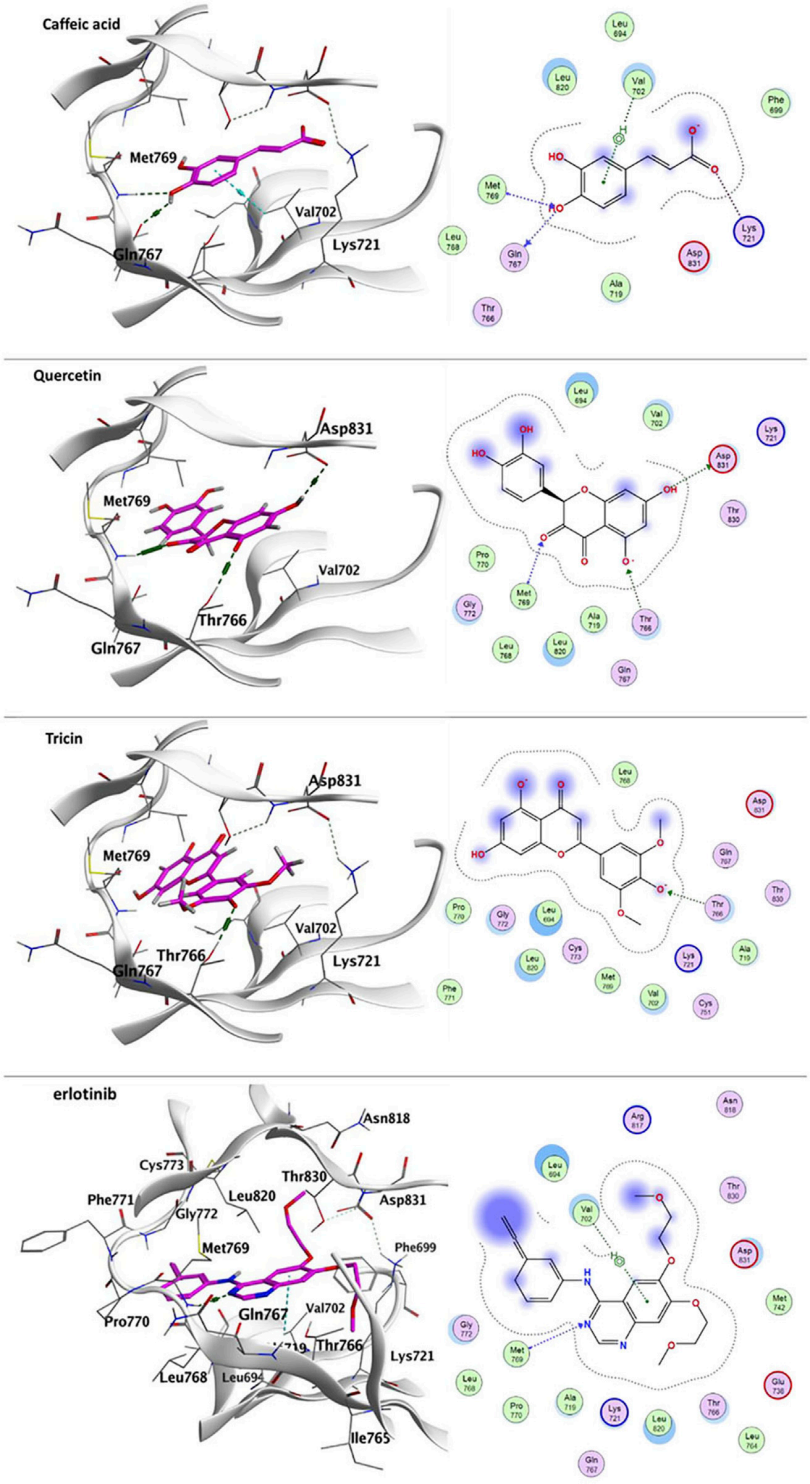
Ligand-protein binding interaction: 3D interactoin diagram of caffeic acid, quercetin and tricin, respectively with EGFR, caffeic acid, quercetin and tricin are represented as purple thick sticks, EGFR amino acid residues of the active site are shown as thin sticks. 2D interaction diagram of caffeic acid, quercetin and tricin, respectively on EGFR (PDB: 1M17). In addition to erlotinib-EGFR diagram.

Docking of caffeic acid, quercetin, tricin, quinic acid, Kaempferin, 3,11,17-trihydroxytricyclo [12.3.1.1^2,6^]- nonadeca-1(18), 2(19), 3,5,14,16-hexaen-8-one (THTCNH), gallic acid, glucocaffeic acid, juglalin and coumaroyl quinic acid into EGFR (PDB: 1M17) showed good docking energy scores ranging from 7.55773878- to 4.85549593- Kcal/mol which are reasonable in comparison with erlotinib; the co-crystallized ligand (Table 5). It was noticed that the docked components showed hydrophobic interaction with Leu694, Val702, Ala719, Lys721, Pro770, Gly772, Thr766, Leu820, Thr830 and Asp831 residues. Caffeic acid exhibited numerous interactions within the EGFR binding pocket, where one hydroxyl group showed two H-bond interactions with Gln767 and Met769 residues. Its phenyl ring made *pi*-H interaction with Val702, and carboxylate showed ionic-bond interaction with Lys721. Chromen-4-one ring allowed quercetin to be deeply oriented with the binding pocket: showing three H-bond interactions with Thr766, Met769 and Asp831. Tricin exhibited H-bond interaction with Thr766 (Figure 5).

**TABLE 5:** Docking details of the selected components of CGBRE on EGFR.

Component	S Score kcal/mol	H-bond interactions	Ionic interaction	*Pi*-H interactions
Caffeic acid	5.22,709,751-	Gln767 Met769	Lys721	Val702
Quercetin	6.30,024,815-	Thr766, Met769, Asp831	-	-
Tricin	6.66,325,808-	Thr766	-	-
Quinic acid	4.85,549,593-	Asp831	-	-
Kaempferin	7.55,773,878-	Met769, Asp831	Lys721	-
THTCNH (3,11,17-trihydroxytricyclo [12.3.1.1^2,6^]-nonadeca-1(18),2(19),3,5,14,16-hexaen-8-one)	5.86,635,351-	Gln767, Asp831	-	-
Gallic acid	5.03,767,824-	Asp831	-	-
Glucocaffeic acid	6.90,880,108-	Gln767, Asp831	-	-
Juglalin	7.39,132,309-	Met769, Asp831	Lys721	-
Coumaroyl quinic acid	7.01,915,741-	Met742, Thr766, Asp831	-	Leu694
Erlotinib	7.73,281,908-	Val702, Met769	-	-

#### 3.3.2 Docking with LRRK2

**FIGURE 6:**
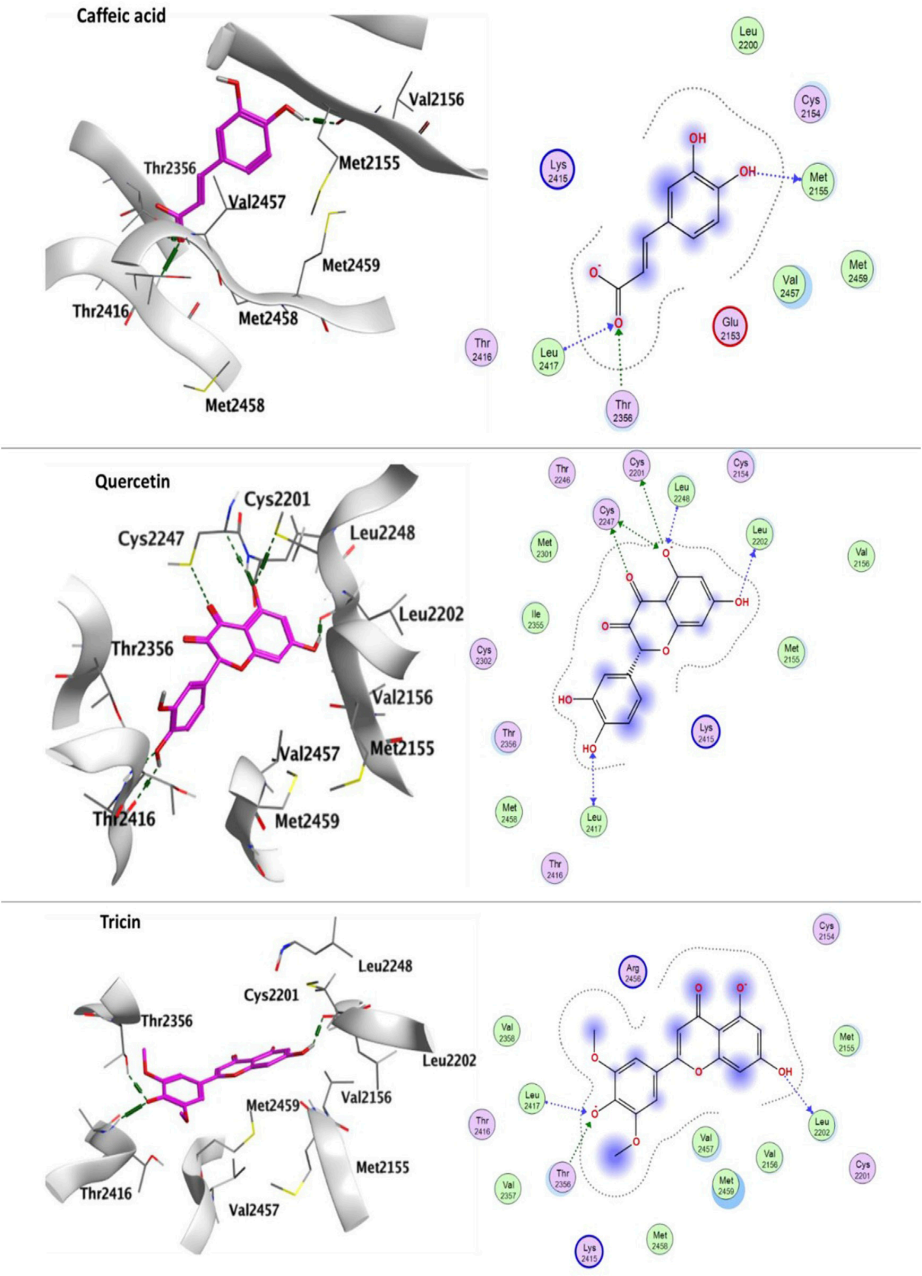
Ligand-protein binding interaction: 3D interactoin diagram of caffeic acid, quercetin and tricin, respectively with LRRK2, caffeic acid, quercetin and tricin are represented as purple thick sticks, LRRK2 amino acid residues of the active site are shown as thin sticks. 2D interaction diagram of caffeic acid, quercetin and tricin, respectively on LRRK2 (PDB: 6DLO).

Leucine-rich repeat serine/threonine-protein kinase 2; LRRK2 (PDB: 6DLO) binding pocket is composed of the following amino acid residues: Val2152, Glu2153, Cys2154, Met2155, Leu2200, Cys2201, Leu2202, Thr2246, Cys2247, Leu2248, Tyr2249, Met2301, Cys2302, Leu2303, Ser2304, Glu2305, Ser2306, Thr2307, Ile2355, Thr2356, Val2357, Val2358, Val2359, Asp2360, Thr2361, Gln2368, Lys2378, Lys2415, Thr2416, Leu2417, Cys2418, Leu2419, Gln2420, Lys2421, Asn2422, Val2455, Arg2456, Val2457, Met2458, Met2459, Thr2460, Asn2468, Met2470 and Ile2498. LRRK2 is a complex protein that consists of multiple domains, including predicted C-terminal WD40 repeats (Piccoli et al., 2014). In our present study, we performed molecular docking on WD40 domain as the seven-bladed WD repeat region is critical for synaptic vesicle trafficking and mediates interaction with multiple vesicle-associated presynaptic proteins (Piccoli et al., 2014). Additionally, it mediates homodimerization and regulates kinase activity of LRRK2 (Zhang et al., 2019a). The binding pocket of LRRK2 was defined by the geometrical approach of MOE Site Finder. Docking of caffeic acid, quercetin, tricin, quinic acid, Kaempferin, 3,11,17-trihydroxytricyclo [12.3.1.1^2,6^]-nonadeca-1(18),2(19),3,5,14,16-hexaen-8-one (THTCNH), gallic acid, glucocaffeic acid, juglalin and coumaroyl quinic acid into LRRK2 showed good docking energy scores ranging from 6.79601002- to −4.32252932 kcal/mol (Table 6). One hydroxyl group of caffeic acid formed H-bond interaction with Met2155 and on the other side carboxylate formed two H-bond interactions with Thr2356 and Leu2417. Quercetin exhibited numerous H-bond interactions with the following amino acid residues: Cys2201, Leu2202, Cys2247, Leu2248 and Leu2417. Tricin formed three H-bond interactions with Leu2202, Thr2356 and Leu2417 (Figure 6).

**TABLE 6:** Docking details of the selected components of CGBRE on LRRK2.

Component	S Score kcal/mol	H-bond interactions
Caffeic acid	4.32,252,932-	Met2155, Thr2356, Leu2417
Quercetin	5.69,858,027-	Cys2201, Leu2202, Cys2247, Leu2248, Leu2417
Tricin	5.50,540,495-	Leu2202, Thr2356, Leu2417
Quinic acid	4.49,469,376-	Met2155, Leu2202, Cys2247
Kaempferin	6.40,560,246-	Leu2202
THTCNH (3,11,17-trihydroxytricyclo [12.3.1.1^2,6^]-nonadeca-1(18),2(19),3,5,14,16-hexaen-8-one)	5.75,019,693-	Cys2201, Leu2202, Met2459, Ile2355
Gallic acid	4.41,518,545-	Leu2417
Glucocaffeic acid	6.09,174,919-	Cys2201, Leu2248, Leu2303, Leu2419, Met2458
Juglalin	6.79,601,002-	Leu2200, Leu2202, Leu2248
Coumaroyl quinic acid	5.93,564,558-	Cys2201, Leu2202, Leu2303, Cys2247, Leu2417

#### 3.3.3 Docking with heat shock protein HSP 90-Alpha

**FIGURE 7:**
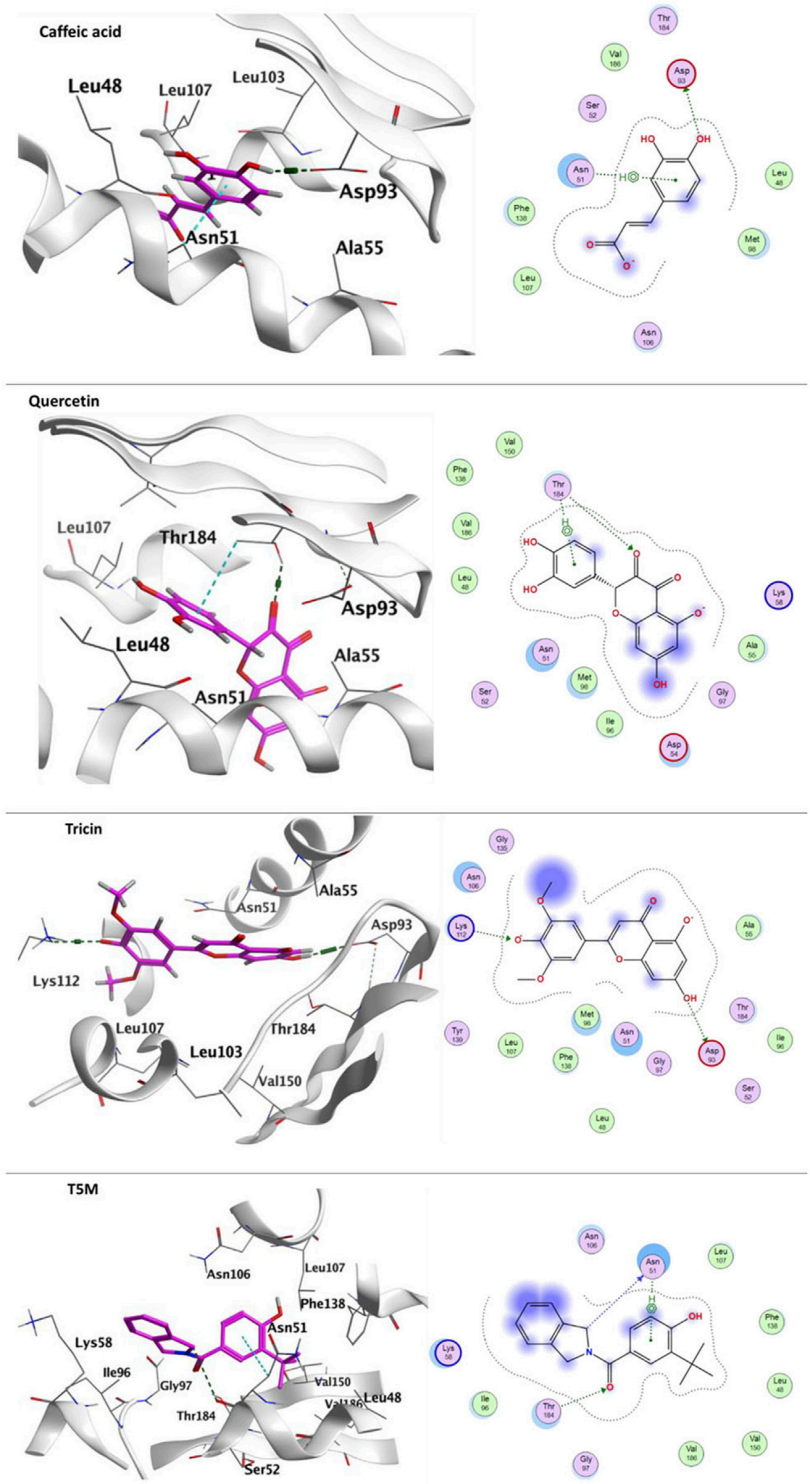
Ligand-protein binding interaction: 3D interactoin diagram of caffeic acid, quercetin and tricin, respectively with HSP90AA1, caffeic acid, quercetin and tricin are represented as purple thick sticks, HSP90AA1 amino acid residues of the active site are shown as thin sticks. 2D interaction diagram of caffeic acid, quercetin and tricin, respectively on HSP90AA1 (PDB: 2XHX). In addition to T5M- HSP90AA1 diagram.

Previous studies have suggested that compounds containing resorcinol may act as inhibitors of HSP90, as reported by Murray et al. (2010) and Woodhead et al. (2010). In this study, docking experiments were conducted with various compounds, including caffeic acid, quercetin, tricin, quinic acid, Kaempferin, 3,11,17-trihydroxytricyclo [12.3.1.1^2,6^]-nonadeca1- (18),2 (19),3,5,14,16-hexaen-8-one (THTCNH), gallic acid, glucocaffeic acid, juglalin and coumaroyl quinic acid, to determine their potential binding affinity with the adenine-binding site of HSP90-AA1 (PDB: 2XHX). The findings showed promising binding affinity, with docking energy scores ranging from 7.22095299 to −4.79,563,427 kcal/mol (Table 7). The components of *C. glauca* were found to exhibit hydrogen-bond interactions as well as *pi*-H bond interactions with the amino acid residues. Notably, one hydroxyl group of caffeic acid displayed H-bond interaction with the acidic Asp93 residue, while its phenyl ring formed arene-H bond interaction with Asn51. Quercetin, on the other hand, formed both H-bond and arene-H bond interactions with Thr184. Tricin demonstrated H-bond interactions with both the acidic Asp93 and the basic Lys112, as depicted in Figure 7.

**TABLE 7:** Docking details of the selected components of CGBRE on HSP90AA1.

Component	S Score kcal/mol	H-bond interactions	*Pi*-H interactions
Caffeic acid	4.80,119,801-	Asp93	Asn51
Quercetin	5.98,078,871-	Thr184	Thr184
Tricin	6.07,966,185-	Asp93, Lys112	-
Quinic acid	5.04,545,879-	Thr184	-
Kaempferin	7.22095299-	Thr184	Asn106
THTCNH (3,11,17-trihydroxytricyclo [12.3.1.1^2,6^]-nonadeca-1(18),2(19),3,5,14,16-hexaen-8-one)	6.7,371,726-	Asn51, Asp93	Thr184
Gallic acid	4.79,563,427-	Ser52, Thr184	-
Glucocaffeic acid	6.4,112,587-	Ser52	Asn51
Juglalin	7.04,507,017-	Asn51, Ser52	-
Coumaroyl quinic acid	6.76,124,716-	Met98, Thr184	-
2- Tert-butyl-4-(1,3-dihydro-2h-isoindol-2-ylcarbonyl)phenol (T5M)	−6.4559	THR184	ASN51

Overall, molecular docking of CGBRE components into the targeted proteins revealed that they exhibited promising binding affinities.

### 3.4 Validation in experimental *in vivo* UC model

**FIGURE 8:**
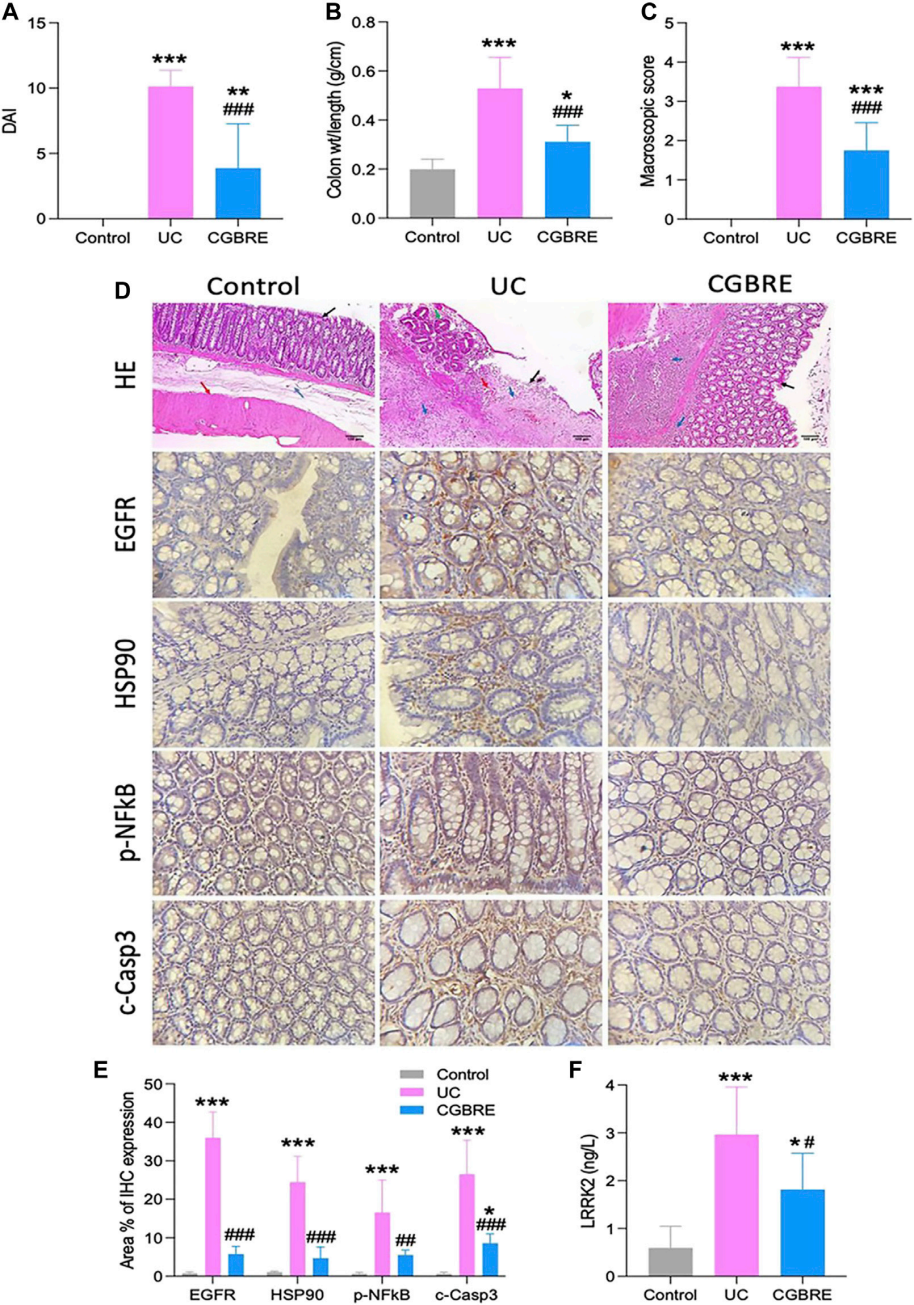
Validation of the effectiveness of CGBRE in a rat model of colitis. **(A).** Disease Activity Index (DAI) (n = 8). **(B)**. Colon weight/length ratio (n = 8). **(C)**. Macroscopic evaluation score (n = 8). **(D)**. Representative images of histological and immunohistochemical examination, in H and E (HE) stained sections, control group had normal colon showing average sized colonic mucosa (black arrow) with underlying submucosa (blue arrow) and muscular layer (red arrow), colons from UC group showed ulceration covered by granulation tissue formation (black arrow) showing congested blood vessels (red arrow) and chronic inflammation (blue arrows) surrounded by dysplastic colonic glands (hyperchromatsia and partial loss of mucin) [green arrow], Treated colon showed complete healing of the ulcer with hyperplasia of glands (black arrow) with sub-mucosal moderate chronic inflammatory cells infiltrate (blue arrows) (H&E X 100). **(E)**. Morphometric analysis of immunohistochemical data (n = 8). **(F)**. LRRK2 (n = 8). Data are expressed as mean ± SD (n = 6–8), *p < 0.05 vs control, **p < 0.01 vs control, ***p < 0.001 vs control, #*p* < 0.05 vs UC, ##*p* < 0.01 vs UC, and ###*p* < 0.001 vs UC. Abbreviations: c-Casp3, cleaved caspase 3; CGBRE, *Casuarina glauca* branchlets ethanolic extract; EGFR, epidermal growth factor receptor; HE, hematoxylin and eosin; HSP90, heat shock protein 90; LRRK2, leucine-rich repeat serine/threonine-protein kinase 2; pNFkB, P65 nuclear factor kappa B; UC, ulcerative colitis, wt, weight.

Having established the effect of CGBRE computationally, its activity using a rat model of UC was assessed. Rats with UC showed high DAI, colon weight/length ratio and macroscopic evaluation scores in addition to histopathological injury of the colon while treated rats showed significantly reduced scores and alleviated tissue damage (Figure 8). Colons from UC rats had high expression of the inflammatory, pNFkB, and the apoptotic, c-Caspase 3 while colons from rats treated with CGBRE had lower expression of both markers (Figure 8).

These findings revealed that CGBRE is effective in treating UC in rats. Consequently, this observed effect was evaluated to find out whether it can be attributed to its activity on the top targets for UC that were identified through *in silico* analysis. Consequently, an assessment of the colon expression of the top three targets (EGFR, LRRK2 and HSP90) was carried out. Indeed, the expression of these proteins was elevated in UC rats while CGBRE treatment significantly reduced their expression (Figure 8).

## 4 Discussion

In this study we have characterized the chemical composition of CGBRE by LC-ESI-MS/MS, separated and identified 86 phytochemical compounds from different classes. Utilizing network analysis enabled us to identify 171 targets in UC that may be targeted by the plant major bioactive compounds. Furthermore, we have recognized the key potential compounds that may elicit a beneficial therapeutic action in UC. Molecular docking studies on some of these compounds displayed their binding with the key identified UC targets. Experimental studies in a rat model of UC confirmed the computational findings by showing a therapeutic action of CGBRE and emphasized the proposed mechanism of action.

Ulcerative colitis (UC) is one of the two major inflammatory bowel diseases that is characterized by relapsing inflammatory conditions, and is considered a defined risk of colorectal cancer with prolonged time (Kvorjak et al., 2020). As the current medications are highly toxic upon long-term treatment and cause severe side effects, it is an urgent to discover new, potent and safe pharmacological approaches for inflammatory bowel diseases as ulcerative colitis (UC) (Baliga et al., 2014). Therefore, herbal medicine is taken into consideration as a complementary and alternative option.

LC-ESI-MS/MS is considered an important tool to characterize different plant extracts to illustrate their phytochemicals according to their pseudo-molecular ion peaks and fragmentation pattern. In line with literature data about the genus *Causarina* (Saleh and El-Lakany, 1979; Gouveia and Castilho, 2012; Salem et al., 2013; Xu et al., 2022), phenolic acids, their derivatives, flavonoid aglycones, their glycosides (mainly flavonol glycosides as quercetin and kaempferol glycosides), cyclic and non-cyclic diarylheptanoid and their glycosides prevailed in CGBRE. Consequently, the potent effects exhibited by CGBRE in the current study may be attributed to synergism effect of these polyphenols.

Concerning the therapeutic effect of phenolic acids in UC, caffeic acid which is one of the predominating phenolic acids in extract, effectively protected against colonic inflammation by decreasing pro-inflammatory cytokines and malondialdehyde levels and increasing antioxidant enzymes (Wan et al., 2021). Gallic acid ameliorated UC in mice by attenuating colonic morphological injury, and decreasing the production of inflammatory cytokines as IL-33, TNF-α, and IFN-y (Yu et al., 2023).

Flavonoids (aglycones/glycosides) have been shown to be effective in both acute and chronic intestinal inflammatory conditions including UC through several mechanisms. The latter include antioxidant activity, inhibition of different enzymes, and conservation of epithelial barrier function, besides their immunomodulatory effects in the gut. Several studies illustrated that both flavonoid aglycones (quercetin, catechin) and glycosides (quercitrin, rutin) possessed intestinal anti-inflammatory activity but glycosylated derivatives showed more potent activity on colonic inflammation as the aglycones are absorbed through small intestine while glycosides reach large intestine where they hydrolyzed to give the aglycone, with quercitrin showed the highest activity (Vezza et al., 2016).

Several Pharmacological studies on experimental animals suggested that quercetin, kaempferol, and rutin are effective in either preventing and/or improving UC complications through different mechanisms. Indeed, treatment with quercetin blocked the increase in colonic malondialdehyde and inhibited the activities of nitric oxide synthase and alkaline phosphatase. Regarding the therapeutic effect of kaempferol in UC, it was shown to ameliorate colitis in mice via a decrease in plasma Leukotriene B4 (LTB4), nitric oxide and PGE2 levels besides upregulation of TFF3 mRNA level. With respect to rutin, biochemical studies illustrated that it enhanced colonic glutathione and protected against ROS in the colon (Baliga et al., 2014). The flavone, tricin, improved chemically-induced colitis in mice by production in lipopolysaccharides-activated cells and myeloperoxidase activity and regulating gut microbiota profile (Li et al., 2021).

Diarylheptanoids are complex polyphenols, having two aromatic rings linked with seven carbon chains and they are structurally diverse. They have several therapeutic effects including anti-inflammatory and anti-ulcer activities (Ganapathy et al., 2019). Several diarylheptanoids possess potential therapeutic benefits on UC as curcumin through reducing the expression of inflammatory cytokines and improving the integrity of intestinal epithelial barrier (Zhong et al., 2023).

Network pharmacology is an exciting approach that help reveal the mechanisms of action of herbal extracts (Mohamed et al., 2022; Elbatreek et al., 2023). Using this approach facilitated the identification of key potential targets of CGBRE in UC, and the top three included EGFR, LRRK2 and HSP90.

The EGFR is a tyrosine kinase receptor that regulates several processes including cell proliferation, differentiation, division, survival, and cancer development (Herbst, 2004; Sabbah et al., 2020). The EGFR protein is composed of two distinct domains, with the N-terminal domain primarily consisting of β-strands and a single α-helix, while the larger C-terminal domain is mainly composed of α-helices. These two domains are separated by a cleft, which has been found to bind ATP, ATP analogues, and ATP inhibitors in previous studies (Stamos et al., 2002). Previous experimental studies of colitis showed that EGFR may regulate inflammatory cytokine production (Lu et al., 2014; El Mahdy et al., 2023), and that the EGFR inhibitor, erlotinib, protects from chronic inflammation and development of dysplasia (Pagán et al., 2011). However, some studies show that EGFR activation may also protect from colitis-associated cancer (Dubé et al., 2012; Dubé et al., 2018). Clinical data show that the expression of EGFR in colonic mucosa is increased in both UC and colon cancer patients (Malecka-Panas et al., 1997). In our study, we only assessed the effects of CGBRE in an acute UC model, thus the long-term effects especially on cancer development necessitates further studies.

The LRRK2 is a kinase whose mutations have been associated with the development of Parkinson’s disease (Li et al., 2014; Nguyen et al., 2020). LRRK2 is also a major susceptibility gene for Crohn’s disease and increased gut inflammations (Liu and Lenardo, 2012; Hui et al., 2018; Herrick and Tansey, 2021). Structurally, the LRRK2 is a complex protein that contains multiple domains, including a Ras of complex domain and a kinase domain, which exhibit both GTPase and kinase activities (Zhang et al., 2019a). Recent data show that LRRK2 polymorphism is linked to the susceptibility of UC, and LRRK2 activates Dectin-1 signaling pathway in UC patients (Sharifinejad et al., 2021). Genetically modified mice with overexpression of LRRK2 or knocking it out show that LRRK2 is a key protein in the inflammatory response in colitis (Takagawa et al., 2018; Yan et al., 2022; Cabezudo et al., 2023). These data suggest that inhibiting LRRK2 may be a potential strategy for treating UC. The effect of CGBRE on LRRK2 observed in our study should be assessed on chronic models of UC and Parkinson’s disease.

The HSP90 is a molecular chaperone that possesses several physiological functions and associates with many signaling pathways, and thus became an attractive therapeutic target for multiple indications (Jackson, 2013). Additionally, HSP90A is a homodimeric protein that comprises three domains: the N-terminal domain, M-domain, and C-terminal domain. The N-terminal domain is considered the catalytic domain that binds with ATP (Pratt and Toft, 2003). HSP90 has been linked to the pathogenesis of UC and its expression is elevated in UC patients (Tomasello et al., 2011; Hoter and Naim, 2019). Indeed, several experimental studies showed beneficial effects upon inhibiting HSP90 in colitis due to interfering with multiple signaling including autophagy and inflammations (Collins et al., 2013; Hoter and Naim, 2019; Yang et al., 2020; Shaaban et al., 2022; Yuan et al., 2022). Here, we showed that CGBRE downregulates colonic HSP90 and thus whether this effect could be favorable in other indications including cancer warrants further investigations.

Molecular docking is an essential bioinformatic tool that utilizes computational methods to predict the binding affinity between a ligand and a targeted protein (Pinzi and Rastelli, 2019). Scoring and ranking ligands on the basis of docking affinity scoring functions (kcal/mol) allow investigators to prioritize the compounds for further acquisition and investigation (Meng et al., 2011). Furthermore, it was reported that a low energy complex and a compatible ligand can lead to potent activity (Agarwal and Mehrotra, 2016). Consequently, the recent molecular docking studies have demonstrated promising outcomes in terms of the binding affinity between CGBRE components and three targeted proteins, namely, EGFR, LRRK2, and HSP90A, which aligned with previous investigations conducted on these proteins (Mohamed et al., 2022; Omena-Okpowe et al., 2022). These findings suggest that CGBRE may be effective in the prevention or treatment of ulcerative colitis mediated by these proteins.

This study focused only on the top three targets as the key proteins that may be responsible for the therapeutic potential of CGBRE in UC, however, other top targets identified by network analysis may also contribute to this effect. For example, P53, estrogen receptor 1 (ESR1) and Cystic Fibrosis Transmembrane Conductance Regulator (CFTR) which are present among the top targets of CGBRE have been shown to be associated to the pathogenesis of UC (Matsuda et al., 1996; Lohi et al., 2002; Arasaradnam et al., 2010; Kobayashi et al., 2017; Villeda-Ramírez et al., 2021). Investigating the role of targeting these proteins and other targets in UC would be interesting.

## 5 Conclusion

By combining computational and experimental analyses, our research has provided valuable insights into the therapeutic potential of CGBRE for UC. The key chemical components of CGBRE have been identified, and its mechanism of action has been explored. Additionally, the findings have demonstrated significant molecular interactions within the ligand binding domain of the target proteins, further supporting its effectiveness in treating UC. Furthermore, CGBRE has been observed to alleviate symptoms of acetic acid-induced UC in rats. However, further preclinical and clinical trials are necessary to confirm the safety and efficacy of CGBRE in UC cases and to evaluate any potential long-term complications.

## Data Availability

The data presented in the study are deposited in the Metabolights repository, accession number MTBLS9078, URL: www.ebi.ac.uk/metabolights/MTBLS9078.

## References

[B1] Abd Al Haleem E. N. Ahmed S. F. Temraz A. El-Tantawy W. H. (2022). Evaluation of the cardioprotective effect of casuarina suberosa extract in rats. Drug Chem. Toxicol. 45, 367–377. 10.1080/01480545.2019.1696815 31778078

[B2] Abdelaziz S. Al Yousef H. M. Al-Qahtani A. S. Hassan W. H. Fantoukh O. I. El-Sayed M. A. (2020). Phytochemical profile, antioxidant and cytotoxic potential of *Parkinsonia aculeata* L. growing in Saudi Arabia. Saudi Pharm. J. 28, 1129–1137. 10.1016/j.jsps.2020.08.001 32922145 PMC7474181

[B3] Aboulthana W. M. K. Refaat E. Khaled S. E. Ibrahim N.E.-S. Youssef A. M. (2022). Metabolite profiling and biological activity assessment of *Casuarina equisetifolia* bark after incorporating gold nanoparticles. Asian Pac. J. Cancer Prev. 23, 3457–3471. 10.31557/APJCP.2022.23.10.3457 36308372 PMC9924326

[B4] Ağalar H. G. Çiftçi G. A. Göğer F. Kırımer N. (2018). Activity guided fractionation of Arum italicum miller tubers and the LC/MS-MS profiles.

[B5] Agarwal C. Hofmann T. Vršanská M. Schlosserová N. Visi-Rajczi E. Voběrková S. (2021). *In vitro* antioxidant and antibacterial activities with polyphenolic profiling of wild cherry, the European larch and sweet chestnut tree bark. Eur. Food Res. Technol. 247, 2355–2370. 10.1007/s00217-021-03796-w

[B6] Agarwal S. Mehrotra R. (2016). An overview of molecular docking. JSM Chem. 4, 1024–1028. 10.47739/2334-1831/1024

[B7] Alatab S. Sepanlou S. G. Ikuta K. Vahedi H. Bisignano C. Safiri S. (2020). The global, regional, and national burden of inflammatory bowel disease in 195 countries and territories, 1990–2017: a systematic analysis for the Global Burden of Disease Study 2017. Lancet gastroenterology hepatology 5, 17–30. 10.1016/S2468-1253(19)30333-4 31648971 PMC7026709

[B8] Alberti A. Riethmüller E. Béni S. (2018). Characterization of diarylheptanoids: an emerging class of bioactive natural products. J. Pharm. Biomed. Analysis 147, 13–34. 10.1016/j.jpba.2017.08.051 28958734

[B9] Al-Hadad A. Al-Zubaidy F. H. (2022). Antibacterial efficacy of Casuarina cunninghamiana extracts against some pathogenic bacteria. Med. Sci. J. Adv. Res. 3, 20–25. 10.46966/msjar.v3i1.35

[B10] Al-Snafi A. E. (2015). The pharmacological importance of Casuarina equisetifolia-An Overview. Int. J. Pharmacol. Screen. Methods 5, 4–9.

[B11] Al-Snafi A. E. (2016). Beneficial medicinal plants in digestive system disorders (part 2): plant based review. IOSR J. Pharm. 6, 85–92. 10.9790/3013-067038592

[B12] Al-Snafi A. E. (2018). Arabian medicinal plants for the treatment of intestinal disorders-plant based review (part 1). health 21, 22.

[B13] Al-Yousef H. M. Abdelaziz S. Hassan W. H. El-Sayed M. A. (2020). Phytochemical and biological characterization of Tephrosia nubica boiss. Growing in Saudi Arabia. Arabian J. Chem. 13, 9216–9230. 10.1016/j.arabjc.2020.11.006

[B14] Arasaradnam R. P. Khoo K. Bradburn M. Mathers J. C. Kelly S. B. (2010). DNA methylation of ESR-1 and N-33 in colorectal mucosa of patients with ulcerative colitis (UC). Epigenetics 5, 422–426. 10.4161/epi.5.5.11959 20505342

[B15] Attia H. G. Albarqi H. A. Said I. G. Alqahtani O. Raey M. a.E. (2022). Synergistic effect between amoxicillin and zinc oxide nanoparticles reduced by oak gall extract against *Helicobacter pylori* . Molecules 27, 4559. 10.3390/molecules27144559 35889432 PMC9320066

[B16] Baliga M. S. Saxena A. Kaur K. Kalekhan F. Chacko A. Venkatesh P. (2014). “Polyphenols in the prevention of ulcerative colitis: past, present and future,” in Polyphenols in human health and disease (Amsterdam, Netherlands: Elsevier), 655–663.

[B17] Ben-Horin S. Kopylov U. Salomon N. (2022). Curcumin-QingDai combination as treatment for moderate-severe ulcerative colitis. Case Rep. Gastroenterol. 16, 563–568. 10.1159/000526646 36824698 PMC9941783

[B18] Borges G. Degeneve A. Mullen W. Crozier A. (2010). Identification of flavonoid and phenolic antioxidants in black currants, blueberries, raspberries, red currants, and cranberries. J. Agric. food Chem. 58, 3901–3909. 10.1021/jf902263n 20000747

[B19] Broom O. J. Widjaya B. Troelsen J. Olsen J. Nielsen O. H. (2009). Mitogen activated protein kinases: a role in inflammatory bowel disease? Clin. Exp. Immunol. 158, 272–280. 10.1111/j.1365-2249.2009.04033.x 19793335 PMC2792822

[B20] Burley S. K. Bhikadiya C. Bi C. Bittrich S. Chao H. Chen L. (2022). RCSB Protein Data bank: tools for visualizing and understanding biological macromolecules in 3D. Protein Sci. 31, e4482. 10.1002/pro.4482 36281733 PMC9667899

[B21] Cabezudo D. Tsafaras G. Van Acker E. Van Den Haute C. Baekelandt V. (2023). Mutant LRRK2 exacerbates immune response and neurodegeneration in a chronic model of experimental colitis. Acta Neuropathol. 146, 245–261. 10.1007/s00401-023-02595-9 37289222 PMC10328902

[B22] Cai Z. Wang S. Li J. (2021). Treatment of inflammatory bowel disease: a comprehensive review. Front. Med. (Lausanne) 8, 765474. 10.3389/fmed.2021.765474 34988090 PMC8720971

[B23] Cartabia A. Tsiokanos E. Tsafantakis N. Lalaymia I. Termentzi A. Miguel M. (2021). The arbuscular mycorrhizal fungus Rhizophagus irregularis MUCL 41833 modulates metabolites production of Anchusa officinalis L. under semi-hydroponic cultivation. Front. plant Sci. 12, 1766. 10.3389/fpls.2021.724352 PMC8443025 34539717

[B24] Chang J. B. Lane M. E. Yang M. Heinrich M. (2016). A hexa-herbal TCM decoction used to treat skin inflammation: an LC-MS-based phytochemical analysis. Planta Medica 82, 1134–1141. 10.1055/s-0042-108206 27272397

[B25] Chin C.-H. Chen S.-H. Wu H.-H. Ho C.-W. Ko M.-T. Lin C.-Y. (2014). cytoHubba: identifying hub objects and sub-networks from complex interactome. BMC Syst. Biol. 8, S11. 10.1186/1752-0509-8-S4-S11 25521941 PMC4290687

[B26] Chonglu Z. Yong Z. Yu C. Zhen C. Qingbin J. Pinyopusarerk K. (2015). “Potential Casuarina species and suitable techniques for the GGW,” in Le projet majeur africain de la Grande Muraille Verte. Marseille: IRD Éditions, 163.

[B27] Christaki S. Bouloumpasi E. Lalidou E. Chatzopoulou P. Irakli M. (2022). Bioactive profile of distilled solid by-products of rosemary, Greek sage and spearmint as affected by distillation methods. Molecules 27, 9058. 10.3390/molecules27249058 36558189 PMC9783801

[B28] Christenhusz M. J. Byng J. W. (2016). The number of known plants species in the world and its annual increase. Phytotaxa 261, 201–217. 10.11646/phytotaxa.261.3.1

[B29] Coccia M. Harrison O. J. Schiering C. Asquith M. J. Becher B. Powrie F. (2012). IL-1β mediates chronic intestinal inflammation by promoting the accumulation of IL-17A secreting innate lymphoid cells and CD4(+) Th17 cells. J. Exp. Med. 209, 1595–1609. 10.1084/jem.20111453 22891275 PMC3428945

[B30] Collins C. B. Aherne C. M. Yeckes A. Pound K. Eltzschig H. K. Jedlicka P. (2013). Inhibition of N-terminal ATPase on HSP90 attenuates colitis through enhanced Treg function. Mucosal Immunol. 6, 960–971. 10.1038/mi.2012.134 23321985 PMC3748235

[B31] Cooper H. S. Murthy S. N. Shah R. S. Sedergran D. J. (1993). Clinicopathologic study of dextran sulfate sodium experimental murine colitis. Lab. Invest. 69, 238–249. 8350599

[B32] Daina A. Michielin O. Zoete V. (2019). SwissTargetPrediction: updated data and new features for efficient prediction of protein targets of small molecules. Nucleic acids Res. 47, W357-W364–W364. 10.1093/nar/gkz382 31106366 PMC6602486

[B33] Diouf D. Sy M. Gherbi H. Bogusz D. Franche C. (2008). “Casuarinaceae,” in «Compendium of transgenic crop plants»: transgenic forest tree species, vol. 9. Editors Kole C. R. Scorza R. Hall T. C. (Oxford, UK: Blackwell Publishing).

[B34] Dubé P. E. Liu C. Y. Girish N. Washington M. K. Polk D. B. (2018). Pharmacological activation of epidermal growth factor receptor signaling inhibits colitis-associated cancer in mice. Sci. Rep. 8, 9119. 10.1038/s41598-018-27353-w 29904166 PMC6002410

[B35] Dubé P. E. Yan F. Punit S. Girish N. Mcelroy S. J. Washington M. K. (2012). Epidermal growth factor receptor inhibits colitis-associated cancer in mice. J. Clin. Invest. 122, 2780–2792. 10.1172/JCI62888 22772467 PMC3408743

[B36] Elbatreek M. H. Fathi A. M. Mahdi I. Abdelfattah M. a.O. Mahmoud M. F. Sobeh M. (2023). Thymus satureioides Coss. combats oral ulcer via inhibition of inflammation, proteolysis, and apoptosis. Inflammopharmacology 31, 2557–2570. 10.1007/s10787-023-01285-y 37477794

[B37] El-Kader A. Mahmoud M. Gharib H. El-Megeed A. Salah A. (2021). Carbon content of some timber trees grown in El-Kassasin Horticulture Research Station. Egypt. J. Agric. Res. 99, 128–135. 10.21608/EJAR.2021.54736.1063

[B38] El Mahdy R. N. Nader M. A. Helal M. G. Abu-Risha S. E. Abdelmageed M. E. (2023). Eicosapentaenoic acid mitigates ulcerative colitis-induced by acetic acid through modulation of NF-κB and TGF-β/EGFR signaling pathways. Life Sci. 327, 121820. 10.1016/j.lfs.2023.121820 37263490

[B39] El-Sayed M. Abbas F. A. Refaat S. El-Shafae A. M. Fikry E. (2021). UPLC-ESI-MS/MS profile of the ethyl acetate fraction of aerial parts of Bougainvillea'Scarlett O'Hara'cultivated in Egypt. Egypt. J. Chem. 64, 793–806. 10.21608/EJCHEM.2020.45694.2933

[B40] El-Shazly M. A. Hamed A. A. Kabary H. A. Ghareeb M. A. (2021). LC-MS/MS profiling, antibiofilm, antimicrobial and bacterial growth kinetic studies of Pluchea dioscoridis extracts. Acta Chromatogr. 34, 338–350. 10.1556/1326.2021.00956

[B41] El-Tantawy W. H. Mohamed S. A. Abd Al Haleem E. N. (2013a). Evaluation of biochemical effects of *Casuarina equisetifolia* extract on gentamicin-induced nephrotoxicity and oxidative stress in rats. Phytochemical analysis. J. Clin. Biochem. Nutr. 53, 158–165. 10.3164/jcbn.13-19 24249970 PMC3818266

[B42] El-Tantawy W. H. Mohamed S. a.-H. Abd Al Haleem E. N. (2013b). Evaluation of biochemical effects of *Casuarina equisetifolia* extract on gentamicin-induced nephrotoxicity and oxidative stress in rats. Phytochemical analysis. J. Clin. Biochem. Nutr. 53, 158–165. 10.3164/jcbn.13-19 24249970 PMC3818266

[B43] El-Tantawy W. H. Saied N. M. (2012). Anti-hepatotoxic effect of Casuarina stricta and Casuarina suberosa extracts on alcohol-induced liver toxicity in rats. Biokemistri 24, 90–96.

[B44] Fathoni A. Saepudin E. Cahyana A. H. Rahayu D. Haib J. (2017). “Identification of nonvolatile compounds in clove (Syzygium aromaticum) from Manado,” in AIP Conference Proceedings, Provo, Utah, USA, July 2017 (Melville, New York: AIP Publishing LLC).030079.

[B45] Felegyi-Tóth C. A. Garádi Z. Darcsi A. Csernák O. Boldizsár I. Béni S. (2022). Isolation and quantification of diarylheptanoids from European hornbeam (Carpinus betulus L.) and HPLC-ESI-MS/MS characterization of its antioxidative phenolics. J. Pharm. Biomed. Analysis 210, 114554. 10.1016/j.jpba.2021.114554 34973466

[B46] Fouad M. R. Salama R. M. Zaki H. F. El-Sahar A. E. (2021). Vildagliptin attenuates acetic acid-induced colitis in rats via targeting PI3K/Akt/NFκB, Nrf2 and CREB signaling pathways and the expression of lncRNA IFNG-AS1 and miR-146a. Int. Immunopharmacol. 92, 107354. 10.1016/j.intimp.2020.107354 33434756

[B47] Ganapathy G. Preethi R. Moses J. Anandharamakrishnan C. (2019). Diarylheptanoids as nutraceutical: a review. Biocatal. Agric. Biotechnol. 19, 101109. 10.1016/j.bcab.2019.101109 32288931 PMC7102868

[B48] Gopichand C. V. Anjani A. Himaja A. Shilpa U. Kumar R. A. Eswaraiah C. M. (2015). Phytochemical evaluation of Lantana camara, *Casuarina equisetifolia*, Michella nilagirica. Indian J. Res. Pharm. Biotechnol. 3, 461.

[B49] Gouveia S. Castilho P. C. (2012). Helichrysum monizii Lowe: phenolic composition and antioxidant potential. Phytochem. Anal. 23, 72–83. 10.1002/pca.1326 21837645

[B50] Gupta M. Mishra V. Gulati M. Kapoor B. Kaur A. Gupta R. (2022). Natural compounds as safe therapeutic options for ulcerative colitis. Inflammopharmacology 30, 397–434. 10.1007/s10787-022-00931-1 35212849 PMC8948151

[B51] Hadjadj S. Esnault M.-A. Berardocco S. Guyot S. Bouchereau A. Ghouini F. (2020). Polyphenol composition and antioxidant activity of Searsia tripartita and Limoniastrum guyonianum growing in Southeastern Algeria. Sci. Afr. 10, e00585. 10.1016/j.sciaf.2020.e00585

[B52] Hamosh A. Scott A. F. Amberger J. S. Bocchini C. A. Mckusick V. A. (2005). Online Mendelian Inheritance in Man (OMIM), a knowledgebase of human genes and genetic disorders. Nucleic acids Res. 33, D514–D517. 10.1093/nar/gki033 15608251 PMC539987

[B53] Han J. Theiss A. L. (2014). Stat3: friend or foe in colitis and colitis-associated cancer? Inflamm. Bowel Dis. 20, 2405–2411. 10.1097/MIB.0000000000000180 25185686 PMC4428549

[B54] Hedl M. Proctor D. D. Abraham C. (2016). JAK2 disease-risk variants are gain of function and JAK signaling threshold determines innate receptor-induced proinflammatory cytokine secretion in macrophages. J. Immunol. 197, 3695–3704. 10.4049/jimmunol.1600845 27664279 PMC5127452

[B55] Herbst R. S. (2004). Review of epidermal growth factor receptor biology. Int. J. Radiat. Oncol. Biol. Phys. 59, 21–26. 10.1016/j.ijrobp.2003.11.041 15142631

[B56] Herrick M. K. Tansey M. G. (2021). Is LRRK2 the missing link between inflammatory bowel disease and Parkinson's disease? NPJ Park. Dis. 7, 26. 10.1038/s41531-021-00170-1 PMC7943592 33750819

[B57] Hossen S. M. Jahidul I. Hossain S. S. Rahman M. M. Firoj A. (2014). Phytochemical and biological evaluation of MeOH extract of *Casuarina equisetifolia* (Linn.) leaves. Eur. J. Med. Plants 4, 927–936. 10.9734/ejmp/2014/9820

[B58] Hoter A. Naim H. Y. (2019). The functions and therapeutic potential of heat shock proteins in inflammatory bowel disease-an update. Int. J. Mol. Sci. 20, 5331. 10.3390/ijms20215331 31717769 PMC6862201

[B59] Hui K. Y. Fernandez-Hernandez H. Hu J. Schaffner A. Pankratz N. Hsu N. Y. (2018). Functional variants in the LRRK2 gene confer shared effects on risk for Crohn's disease and Parkinson's disease. Sci. Transl. Med. 10, eaai7795. 10.1126/scitranslmed.aai7795 29321258 PMC6028002

[B60] Jackson S. E. (2013). Hsp90: structure and function. Top. Curr. Chem. 328, 155–240. 10.1007/128_2012_356 22955504

[B61] Jagtap A. G. Shirke S. S. Phadke A. S. (2004). Effect of polyherbal formulation on experimental models of inflammatory bowel diseases. J. Ethnopharmacol. 90, 195–204. 10.1016/j.jep.2003.09.042 15013181

[B62] Jelača S. Dajić-Stevanović Z. Vuković N. Kolašinac S. Trendafilova A. Nedialkov P. (2022). Beyond traditional use of alchemilla vulgaris: genoprotective and antitumor activity *in vitro* . Molecules 27, 8113. 10.3390/molecules27238113 36500205 PMC9740270

[B63] Jiménez Sánchez C. (2016). Screening of vegetables and derivatives as sources of bioactive compounds: analytical characterization, purification, and evaluation of their *in vitro* bioactivity against metabolic sindrome and related disorders.

[B64] Kaneda N. Kinghorn A. Farnsworth N. Tuchinda P. Udchachon J. Santisuki T. (1990). Two diarylheptanoids and a lignan fromCasuarina junghuhniana. Phytochemistry 29, 3366–3368. 10.1016/0031-9422(90)80220-b

[B65] Kantheti U. K. Kumar D. Ganinna B. Nath P. (2014). *Casuarina equisetifolia* effect as antidiabetic and antihyperlipidemic on streptozocin induced rats with diabetes. IJCTPR 2, 432–436.

[B66] Karar M. E. Quiet L. Rezk A. Jaiswal R. Rehders M. Ullrich M. (2016). Phenolic profile and *in vitro* assessment of cytotoxicity and antibacterial activity of Ziziphus spina-christi leaf extracts. Med. Chem. 6, 143–156. 10.4172/2161-0444.1000339

[B67] Kobayashi K. Tomita H. Shimizu M. Tanaka T. Suzui N. Miyazaki T. (2017). p53 expression as a diagnostic biomarker in ulcerative colitis-associated cancer. Int. J. Mol. Sci. 18, 1284. 10.3390/ijms18061284 28621756 PMC5486106

[B68] Kobayashi T. Siegmund B. Le Berre C. Wei S. C. Ferrante M. Shen B. (2020). Ulcerative colitis. Nat. Rev. Dis. Prim. 6, 74. 10.1038/s41572-020-0205-x 32913180

[B69] Kotlyar M. Pastrello C. Ahmed Z. Chee J. Varyova Z. Jurisica I. (2022). IID 2021: towards context-specific protein interaction analyses by increased coverage, enhanced annotation and enrichment analysis. Nucleic Acids Res. 50, D640–d647. 10.1093/nar/gkab1034 34755877 PMC8728267

[B70] Kulkarni A. V. Anjaneyareddy P. G. S. B. Kamalakannan R. Rajkumar R. (2019). “Genomic selection for economic traits in Casuarina junghuhniana natural population,” in Casuarinas for green economy and environmental sustainability. Editors Haruthaithanasan M. Pinyopusarerk K. Nicodemus A. Bush D. Thomson L. (Krabi, Thailand: Kasetsart Agricultural and Agro-Industrial Product Improvement Institute, Kasetsart University).

[B71] Kvorjak M. Ahmed Y. Miller M. L. Sriram R. Coronnello C. Hashash J. G. (2020). Cross-talk between colon cells and macrophages increases ST6GALNAC1 and MUC1-sTn expression in ulcerative colitis and colitis-associated colon cancer. Cancer Immunol. Res. 8, 167–178. 10.1158/2326-6066.CIR-19-0514 31831633 PMC7002239

[B72] Li C. Seeram N. P. (2018). Ultra‐fast liquid chromatography coupled with electrospray ionization time‐of‐flight mass spectrometry for the rapid phenolic profiling of red maple (Acer rubrum) leaves. J. Sep. Sci. 41, 2331–2346. 10.1002/jssc.201800037 29512337 PMC7167591

[B73] Li C.-W. Dong H.-J. Cui C.-B. (2015a). The synthesis and antitumor activity of twelve galloyl glucosides. Molecules 20, 2034–2060. 10.3390/molecules20022034 25633333 PMC6272398

[B74] Li J. Q. Tan L. Yu J. T. (2014). The role of the LRRK2 gene in Parkinsonism. Mol. Neurodegener. 9, 47. 10.1186/1750-1326-9-47 25391693 PMC4246469

[B75] Li X.-X. Chen S.-G. Yue G.G.-L. Kwok H.-F. Lee J.K.-M. Zheng T. (2021). Natural flavone tricin exerted anti-inflammatory activity in macrophage via NF-κB pathway and ameliorated acute colitis in mice. Phytomedicine 90, 153625. 10.1016/j.phymed.2021.153625 34256329

[B76] Li Y. Liu Y. Liu R. Liu S. Zhang X. Wang Z. (2015b). HPLC-LTQ-orbitrap MS n profiling method to comprehensively characterize multiple chemical constituents in xiao-er-qing-jie granules. Anal. Methods 7, 7511–7526. 10.1039/c5ay00420a

[B77] Liu C. Wahefu A. Lu X. Abdulla R. Dou J. Zhao H. (2022). Chemical profiling of kaliziri injection and quantification of six caffeoyl quinic acids in beagle plasma by LC-MS/MS. Pharmaceuticals 15, 663. 10.3390/ph15060663 35745582 PMC9230828

[B78] Liu Z. Lenardo M. J. (2012). The role of LRRK2 in inflammatory bowel disease. Cell Res. 22, 1092–1094. 10.1038/cr.2012.42 22430149 PMC3391018

[B79] Llorent-Martínez E. Ortega-Barrales P. Zengin G. Uysal S. Ceylan R. Guler G. (2016). Lathyrus aureus and Lathyrus pratensis: characterization of phytochemical profiles by liquid chromatography-mass spectrometry, and evaluation of their enzyme inhibitory and antioxidant activities. RSC Adv. 6, 88996–89006. 10.1039/c6ra17170b

[B80] Llorent-Martínez E. J. Gouveia S. Castilho P. C. (2015). Analysis of phenolic compounds in leaves from endemic trees from Madeira Island. A contribution to the chemotaxonomy of Laurisilva forest species. Industrial Crops Prod. 64, 135–151. 10.1016/j.indcrop.2014.10.068

[B81] Lohi H. Mäkelä S. Pulkkinen K. Höglund P. Karjalainen-Lindsberg M. L. Puolakkainen P. (2002). Upregulation of CFTR expression but not SLC26A3 and SLC9A3 in ulcerative colitis. Am. J. Physiol. Gastrointest. Liver Physiol. 283, G567–G575. 10.1152/ajpgi.00356.2001 12181169

[B82] López-Fernández O. Domínguez R. Pateiro M. Munekata P. E. Rocchetti G. Lorenzo J. M. (2020). Determination of polyphenols using liquid chromatography–tandem mass spectrometry technique (LC–MS/MS): a review. Antioxidants 9, 479. 10.3390/antiox9060479 32498428 PMC7346120

[B83] Lu N. Wang L. Cao H. Liu L. Van Kaer L. Washington M. K. (2014). Activation of the epidermal growth factor receptor in macrophages regulates cytokine production and experimental colitis. J. Immunol. 192, 1013–1023. 10.4049/jimmunol.1300133 24391216 PMC4006992

[B84] Maisto M. Piccolo V. Novellino E. Schiano E. Iannuzzo F. Ciampaglia R. (2022). Optimization of phlorizin extraction from annurca apple tree leaves using response surface methodology. Antioxidants 11, 1933. 10.3390/antiox11101933 36290654 PMC9598179

[B85] Malecka-Panas E. Kordek R. Biernat W. Tureaud J. Liberski P. P. Majumdar A. P. (1997). Differential activation of total and EGF receptor (EGF-R) tyrosine kinase (tyr-k) in the rectal mucosa in patients with adenomatous polyps, ulcerative colitis and colon cancer. Hepatogastroenterology 44, 435–440. 9164515

[B86] Mamillapalli V. Chapala R. H. Sareddu T. K. S. Kondaveeti L. S. Pattipati S. Khantamneni P. (2020). Evaluation of phytochemical and *in vitro* anti-inflammatory activity of leaf and fruit extracts of *Casuarina equisetifolia* . Asian J. Pharm. Technol. 10, 143–148. 10.5958/2231-5713.2020.00025.2

[B87] Masullo M. Mari A. Cerulli A. Bottone A. Kontek B. Olas B. (2016). Quali-quantitative analysis of the phenolic fraction of the flowers of Corylus avellana, source of the Italian PGI product “Nocciola di Giffoni”: isolation of antioxidant diarylheptanoids. Phytochemistry 130, 273–281. 10.1016/j.phytochem.2016.06.007 27372151

[B88] Mateescu R. B. Bastian A. E. Nichita L. Marinescu M. Rouhani F. Voiosu A. M. (2017). Vascular endothelial growth factor - key mediator of angiogenesis and promising therapeutical target in ulcerative colitis. Rom. J. Morphol. Embryol. 58, 1339–1345. 29556626

[B89] Matsuda K. Watanabe H. Ajioka Y. Kobayashi M. Saito H. Sasaki M. (1996). Ulcerative colitis with overexpression of p53 preceding overt histological abnormalities of the epithelium. J. Gastroenterol. 31, 860–867. 10.1007/BF02358616 9027653

[B90] Matsuoka K. Hibi T. (2023). Author Correction: etrasimod for ulcerative colitis: evaluating phase III results. Nat. Rev. Gastroenterol. Hepatol. 10.1038/s41575-023-00817-9 37221319

[B91] Mekam P. N. Martini S. Nguefack J. Tagliazucchi D. Stefani E. (2019). Phenolic compounds profile of water and ethanol extracts of Euphorbia hirta L. leaves showing antioxidant and antifungal properties. South Afr. J. Bot. 127, 319–332. 10.1016/j.sajb.2019.11.001

[B92] Mohamed M. E. Tawfeek N. Elbaramawi S. S. Elbatreek M. H. Fikry E. (2022). *Agathis robusta* bark extract protects from renal ischemia-reperfusion injury: phytochemical, *in silico* and *in vivo* studies. Pharmaceuticals 15, 1270. 10.3390/ph15101270 36297382 PMC9610891

[B93] Murray C. W. Carr M. G. Callaghan O. Chessari G. Congreve M. Cowan S. (2010). Fragment-based drug discovery applied to Hsp90. Discovery of two lead series with high ligand efficiency. J. Med. Chem. 53, 5942–5955. 10.1021/jm100059d 20718493

[B94] Muthuraj S. Muthusamy P. Radha R. Ilango K. (2020). Pharmacognostical, phytochemical studies and *in vitro* antidiabetic evaluation of seed extracts of casuarina equisetifolia linn. J. Phytopharm. 9, 410–418. 10.31254/phyto.2020.9605

[B95] Nguyen A. P. T. Tsika E. Kelly K. Levine N. Chen X. West A. B. (2020). Dopaminergic neurodegeneration induced by Parkinson's disease-linked G2019S LRRK2 is dependent on kinase and GTPase activity. Proc. Natl. Acad. Sci. U. S. A. 117, 17296–17307. 10.1073/pnas.1922184117 32631998 PMC7382283

[B96] Olennikov D. N. Vasilieva A. G. Chirikova N. K. (2020). Fragaria viridis fruit metabolites: variation of LC-MS profile and antioxidant potential during ripening and storage. Pharmaceuticals 13, 262. 10.3390/ph13090262 32971880 PMC7559413

[B97] Omena-Okpowe B. Khaled M. K. Akinseye S. P. Hammouda M. A. Bora A. Abayomi M. A. (2022). Bioactive Constituents of Safflower plant as potential inhibitors of mutant Leucine-rich repeat kinase 2 (LRRK2): docking, Pharmacokinetics properties and Molecular Dynamics studies.

[B98] Onkokesung N. Reichelt M. Van Doorn A. Schuurink R. C. Van Loon J. J. Dicke M. (2014). Modulation of flavonoid metabolites in *Arabidopsis thaliana* through overexpression of the MYB75 transcription factor: role of kaempferol-3, 7-dirhamnoside in resistance to the specialist insect herbivore Pieris brassicae. J. Exp. Bot. 65, 2203–2217. 10.1093/jxb/eru096 24619996 PMC3991749

[B99] Osman E. E. Morsi E. A. El-Sayed M. M. Gobouri A. Abdel-Hameed E.-S. S. (2021). Identification of the volatile and nonvolatile constituents of Schinus molle (L.) fruit extracts and estimation of their activities as anticancer agents. J. Appl. Pharm. Sci. 11, 163â€“171. 10.7324/JAPS.2021.110719

[B100] Pagán B. Isidro A. A. Cruz M. L. Ren Y. Coppola D. Wu J. (2011). Erlotinib inhibits progression to dysplasia in a colitis-associated colon cancer model. World J. Gastroenterol. 17, 4858–4866. 10.3748/wjg.v17.i44.4858 22171126 PMC3235628

[B101] Pawar D. S. Nasreen S. (2018). HR-LCMS of phytoconstituents and antifungal activity of medicinal plants. J. Med. Plants 6, 173–176.

[B102] Peyrin-Biroulet L. Danese S. Argollo M. Pouillon L. Peppas S. Gonzalez-Lorenzo M. (2019). Loss of response to vedolizumab and ability of dose intensification to restore response in patients with Crohn's disease or ulcerative colitis: a systematic review and meta-analysis. Clin. Gastroenterol. Hepatol. 17, 838–846. 10.1016/j.cgh.2018.06.026 29935327

[B103] Piccoli G. Onofri F. Cirnaru M. D. Kaiser C. J. Jagtap P. Kastenmüller A. (2014). Leucine-rich repeat kinase 2 binds to neuronal vesicles through protein interactions mediated by its C-terminal WD40 domain. Mol. Cell Biol. 34, 2147–2161. 10.1128/MCB.00914-13 24687852 PMC4054300

[B104] Piñero J. Ramírez-Anguita J. M. Saüch-Pitarch J. Ronzano F. Centeno E. Sanz F. (2020). The DisGeNET knowledge platform for disease genomics: 2019 update. Nucleic acids Res. 48, D845-D855–D855. 10.1093/nar/gkz1021 31680165 PMC7145631

[B105] Pinzi L. Rastelli G. (2019). Molecular docking: shifting paradigms in drug discovery. Int. J. Mol. Sci. 20, 4331. 10.3390/ijms20184331 31487867 PMC6769923

[B106] Pratt W. B. Toft D. O. (2003). Regulation of signaling protein function and trafficking by the hsp90/hsp70-based chaperone machinery. Exp. Biol. Med. 228, 111–133. 10.1177/153537020322800201 12563018

[B107] Rahman R. (2012). Spasmolytic activity of *Casuarina equisetifolia* bark extract. Int. J. Pharm. Sci. Res. 3, 1452.

[B108] Raudvere U. Kolberg L. Kuzmin I. Arak T. Adler P. Peterson H. (2019). g:Profiler: a web server for functional enrichment analysis and conversions of gene lists (2019 update). Nucleic Acids Res. 47, W191-W198–w198. 10.1093/nar/gkz369 31066453 PMC6602461

[B109] Razgonova M. Zakharenko A. Pikula K. Manakov Y. Ercisli S. Derbush I. (2021). LC-MS/MS screening of phenolic compounds in wild and cultivated grapes vitis amurensis rupr. Molecules 26, 3650. 10.3390/molecules26123650 34203808 PMC8232594

[B110] Rebhan M. Chalifa-Caspi V. Prilusky J. Lancet D. (1997). GeneCards: integrating information about genes, proteins and diseases. Trends Genet. TIG 13, 163. 10.1016/s0168-9525(97)01103-7 9097728

[B111] Reed K. A. (2009). Identification of phenolic compounds from peanut skin using HPLC-MSn. Va. Tech. Available at: http://hdl.handle.net/10919/30160 .

[B112] Sabbah D. A. Hajjo R. Sweidan K. (2020). Review on epidermal growth factor receptor (EGFR) structure, signaling pathways, interactions, and recent updates of EGFR inhibitors. Curr. Top. Med. Chem. 20, 815–834. 10.2174/1568026620666200303123102 32124699

[B113] Saber S. Khalil R. M. Abdo W. S. Nassif D. El-Ahwany E. (2019). Olmesartan ameliorates chemically-induced ulcerative colitis in rats via modulating NFκB and Nrf-2/HO-1 signaling crosstalk. Toxicol. Appl. Pharmacol. 364, 120–132. 10.1016/j.taap.2018.12.020 30594690

[B114] Saeed M. M. Fernández-Ochoa A. Saber F. R. Sayed R. H. Cádiz-Gurrea M. D. L. L. Elmotayam A. K. (2022). The potential neuroprotective effect of Cyperus esculentus L. Extract in scopolamine-induced cognitive impairment in rats: extensive biological and metabolomics approaches. Molecules 27, 7118. 10.3390/molecules27207118 36296710 PMC9606906

[B115] Safran M. Dalah I. Alexander J. Rosen N. Iny Stein T. Shmoish M. (2010). GeneCards Version 3: the human gene integrator. Database 2010, baq020. 10.1093/database/baq020 20689021 PMC2938269

[B116] Saleh N. A. El-Lakany M. H. (1979). A quantitative variation in the flavonoids and phenolics of some Casuarina species. Biochem. Syst. Ecol. 7, 13–15. 10.1016/0305-1978(79)90034-6

[B117] Salem M. Z. M. Aly H. Gohar Y. El-Sayed A.-W. (2013). Biological activity of extracts from Morus alba L., Albizzia lebbeck (L.) Benth. and *Casuarina glauca* Sieber against the growth of some pathogenic bacteria. Int. J. Agric. Food Res. 2. 10.24102/ijafr.v2i1.122

[B118] Samet S. Ayachi A. Fourati M. Mallouli L. Allouche N. Treilhou M. (2022). Antioxidant and antimicrobial activities of Erodium arborescens aerial Part Extracts and characterization by LC-HESI-MS2 of its acetone extract. Molecules 27, 4399. 10.3390/molecules27144399 35889269 PMC9318634

[B119] Samuel S. Ingle S. B. Dhillon S. Yadav S. Harmsen W. S. Zinsmeister A. R. (2013). Cumulative incidence and risk factors for hospitalization and surgery in a population-based cohort of ulcerative colitis. Inflamm. Bowel Dis. 19, 1858–1866. 10.1097/MIB.0b013e31828c84c5 23660997 PMC4526131

[B120] Sánchez‐Rabaneda F. Jáuregui O. Lamuela‐Raventós R. M. Viladomat F. Bastida J. Codina C. (2004). Qualitative analysis of phenolic compounds in apple pomace using liquid chromatography coupled to mass spectrometry in tandem mode. Rapid Commun. Mass Spectrom. 18, 553–563. 10.1002/rcm.1370 14978800

[B121] Sanz M. De Simón B. F. Cadahía E. Esteruelas E. Muñoz A. M. Hernández T. (2012). LC‐DAD/ESI‐MS/MS study of phenolic compounds in ash (Fraxinus excelsior L. and *F. americana* L.) heartwood. Effect of toasting intensity at cooperage. J. Mass Spectrom. 47, 905–918. 10.1002/jms.3040 22791259

[B122] Shaaban A. A. Abdelhamid A. M. Shaker M. E. Cavalu S. Maghiar A. M. Alsayegh A. A. (2022). Combining the HSP90 inhibitor TAS-116 with metformin effectively degrades the NLRP3 and attenuates inflammasome activation in rats: a new management paradigm for ulcerative colitis. Biomed. Pharmacother. 153, 113247. 10.1016/j.biopha.2022.113247 35724510

[B123] Shahid M. Raish M. Ahmad A. Bin Jardan Y. A. Ansari M. A. Ahad A. (2022). Sinapic acid ameliorates acetic acid-induced ulcerative colitis in rats by suppressing inflammation, oxidative stress, and apoptosis. Molecules 27, 4139. 10.3390/molecules27134139 35807383 PMC9268465

[B124] Shalini S. Kumar A. (2011). Study on phytochemical profile and Anti-ulcerogenic effect of *Casuarina equisetifolia* (L.). Asian J. Pharm. Sci. Technol. 1, 12.

[B125] Shannon P. Markiel A. Ozier O. Baliga N. S. Wang J. T. Ramage D. (2003). Cytoscape: a software environment for integrated models of biomolecular interaction networks. Genome Res. 13, 2498–2504. 10.1101/gr.1239303 14597658 PMC403769

[B126] Sharifinejad N. Mozhgani S. H. Bakhtiyari M. Mahmoudi E. (2021). Association of LRRK2 rs11564258 single nucleotide polymorphisms with type and extent of gastrointestinal mycobiome in ulcerative colitis: a case-control study. Gut Pathog. 13, 56. 10.1186/s13099-021-00453-1 34593025 PMC8482594

[B127] Shi Y. J. Gong H. F. Zhao Q. Q. Liu X. S. Liu C. Wang H. (2019). Critical role of toll-like receptor 4 (TLR4) in dextran sulfate sodium (DSS)-Induced intestinal injury and repair. Toxicol. Lett. 315, 23–30. 10.1016/j.toxlet.2019.08.012 31442584

[B128] Silva M. R. (2019). Determination of chemical profile of cagaita (Eugenia dysenterica) and its ice cream using paper spray ionization mass spectrometry and headspace solid-phase microextraction combined with gas chromatography-mass spectrometry.

[B129] Silva M. R. Freitas L. G. Souza A. G. Araújo R. L. Lacerda I. C. Pereira H. V. (2019). Antioxidant activity and metabolomic analysis of cagaitas (Eugenia dysenterica) using paper spray mass spectrometry. J. Braz. Chem. Soc. 30, 1034–1044. 10.21577/0103-5053.20190002

[B130] Sogo A. Setoguchi H. Noguchi J. Jaffré T. Tobe H. (2001). Molecular phylogeny of Casuarinaceae based on rbcL and matK gene sequences. J. Plant Res. 114, 459–464. 10.1007/pl00014011

[B131] Spínola V. Pinto J. Castilho P. C. (2015). Identification and quantification of phenolic compounds of selected fruits from Madeira Island by HPLC-DAD–ESI-MSn and screening for their antioxidant activity. Food Chem. 173, 14–30. 10.1016/j.foodchem.2014.09.163 25465990

[B132] Sriram N. (2011). Antidiabetic and antihyperlipidemic activity of bark of *Casuarina equisetifolia* on streptozotocin induced diabetic rats. Int. J. Pharm. Rev. Res. 1, 4–8.

[B133] Stamos J. Sliwkowski M. X. Eigenbrot C. (2002). Structure of the epidermal growth factor receptor kinase domain alone and in complex with a 4-anilinoquinazoline inhibitor. J. Biol. Chem. 277, 46265–46272. 10.1074/jbc.M207135200 12196540

[B134] Szklarczyk D. Gable A. L. Nastou K. C. Lyon D. Kirsch R. Pyysalo S. (2021). The STRING database in 2021: customizable protein–protein networks, and functional characterization of user-uploaded gene/measurement sets. Nucleic acids Res. 49, D605–D612. 10.1093/nar/gkaa1074 33237311 PMC7779004

[B135] Takagawa T. Kitani A. Fuss I. Levine B. Brant S. R. Peter I. (2018). An increase in LRRK2 suppresses autophagy and enhances Dectin-1-induced immunity in a mouse model of colitis. Sci. Transl. Med. 10, eaan8162. 10.1126/scitranslmed.aan8162 29875204 PMC6636639

[B136] Takahashi H. Iuchi M. Fujita Y. Minami H. Fukuyama Y. (1999). Coumaroyl triterpenes from *Casuarina equisetifolia* . Phytochemistry 51, 543–550. 10.1016/s0031-9422(99)00070-9

[B137] Tang J. Dunshea F. R. Suleria H. A. (2019). Lc-esi-qtof/ms characterization of phenolic compounds from medicinal plants (hops and juniper berries) and their antioxidant activity. Foods 9, 7. 10.3390/foods9010007 31861820 PMC7023254

[B138] Tang T. Targan S. R. Li Z. S. Xu C. Byers V. S. Sandborn W. J. (2011). Randomised clinical trial: herbal extract HMPL-004 in active ulcerative colitis - a double-blind comparison with sustained release mesalazine. Aliment. Pharmacol. Ther. 33, 194–202. 10.1111/j.1365-2036.2010.04515.x 21114791

[B139] Tchoumtchoua J. Njamen D. Mbanya J. C. Skaltsounis A. L. Halabalaki M. (2013). Structure‐oriented UHPLC‐LTQ Orbitrap‐based approach as a dereplication strategy for the identification of isoflavonoids from Amphimas pterocarpoides crude extract. J. Mass Spectrom. 48, 561–575. 10.1002/jms.3167 23674281

[B140] Tomasello G. Sciumé C. Rappa F. Rodolico V. Zerilli M. Martorana A. (2011). Hsp10, Hsp70, and Hsp90 immunohistochemical levels change in ulcerative colitis after therapy. Eur. J. Histochem 55, e38. 10.4081/ejh.2011.e38 22297444 PMC3284240

[B141] Verardo G. Duse I. Callea A. (2009). Analysis of underivatized oligosaccharides by liquid chromatography/electrospray ionization tandem mass spectrometry with post‐column addition of formic acid. Rapid Commun. Mass Spectrom. Int. J. Devoted Rapid Dissem. Up‐to‐the‐Minute Res. Mass Spectrom. 23, 1607–1618. 10.1002/rcm.4047 19408275

[B142] Vezza T. Rodríguez-Nogales A. Algieri F. Utrilla M. P. Rodriguez-Cabezas M. E. Galvez J. (2016). Flavonoids in inflammatory bowel disease: a review. nutrients 8, 211. 10.3390/nu8040211 27070642 PMC4848680

[B143] Villeda-Ramírez M. A. Meza-Guillen D. Barreto-Zúñiga R. Yamamoto-Furusho J. K. (2021). ABCC7/CFTR expression is associated with the clinical course of ulcerative colitis patients. Gastroenterol. Res. Pract. 2021, 5536563. 10.1155/2021/5536563 34512749 PMC8426104

[B144] Wan F. Zhong R. Wang M. Zhou Y. Chen Y. Yi B. (2021). Caffeic acid supplement alleviates colonic inflammation and oxidative stress potentially through improved gut microbiota community in mice. Front. Microbiol. 12, 784211. 10.3389/fmicb.2021.784211 34867926 PMC8636926

[B145] Wang P. Wang H. Cai C. Wang H. Kong F. Yuan J. (2020). Three new compounds from the litters of *Casuarina equisetifolia* . Phytochem. Lett. 35, 58–62. 10.1016/j.phytol.2019.10.011

[B146] Wang Y. Chen P. (2022). Combination of HPLC-Q-TOF-MS/MS, network pharmacology, and molecular docking to reveal the mechanism of apple pollen in the treatment of type 2 diabetes mellitus. Evidence-Based Complementary Altern. Med. 2022, 3221196. 10.1155/2022/3221196 PMC9155929 35656465

[B147] Wilson K. Johnson L. (1989). “Casuarinaceae,” in Flora of Australia, vol. 3: hamamelidales to casuarinales. Editor George A. S. (Canberra: Australian Government Publishing Service), 100–174.

[B148] Xiao Y. Liu L. Bian J. Yan C. Ye L. Zhao M. (2018). Identification of multiple constituents in shuganjieyu capsule and rat plasma after oral administration by ultra-performance liquid chromatography coupled with electrospray ionization and ion trap mass spectrometry. Acta Chromatogr. 30, 95–102. 10.1556/1326.2017.00094

[B149] Xu X. Chen L. Luo Y. Gao R. Xu Y. Yang J. (2022). Discovery of cyclic diarylheptanoids as inhibitors against influenza A virus from the roots of *Casuarina equisetifolia* . J. Nat. Prod. 85, 2142–2148. 10.1021/acs.jnatprod.2c00335 36040315

[B150] Yan J. Yu W. Wang G. Lu C. Liu C. Jiang L. (2022). LRRK2 deficiency mitigates colitis progression by favoring resolution of inflammation and restoring homeostasis of gut microbiota. Genomics 114, 110527. 10.1016/j.ygeno.2022.110527 36455749

[B151] Yang M. Zhang F. Yang C. Wang L. Sung J. Garg P. (2020). Oral targeted delivery by nanoparticles enhances efficacy of an Hsp90 inhibitor by reducing systemic exposure in murine models of colitis and colitis-associated cancer. J. Crohns Colitis 14, 130–141. 10.1093/ecco-jcc/jjz113 31168612

[B152] Yu T.-Y. Feng Y.-M. Kong W.-S. Li S.-N. Sun X.-J. Zhou G. (2023). Gallic acid ameliorates dextran sulfate sodium-induced ulcerative colitis in mice via inhibiting NLRP3 inflammasome. Front. Pharmacol. 14, 1095721. 10.3389/fphar.2023.1095721 36762118 PMC9905138

[B153] Yuan Y. Wu H. Shuai B. Liu C. Zhu F. Gao F. (2022). Mechanism of HSP90 inhibitor in the treatment of DSS-induced colitis in mice by inhibiting MAPK pathway and synergistic effect of compound Sophora decoction. Curr. Pharm. Des. 28, 3456–3468. 10.2174/1381612829666221122113929 36415092

[B154] Zhan L. Huang X. Xue J. Liu H. Xiong C. Wang J. (2021). MALDI-TOF/TOF tandem mass spectrometry imaging reveals non-uniform distribution of disaccharide isomers in plant tissues. Food Chem. 338, 127984. 10.1016/j.foodchem.2020.127984 33092001

[B155] Zhang P. Fan Y. Ru H. Wang L. Magupalli V. G. Taylor S. S. (2019a). Crystal structure of the WD40 domain dimer of LRRK2. Proc. Natl. Acad. Sci. U. S. A. 116, 1579–1584. 10.1073/pnas.1817889116 30635421 PMC6358694

[B156] Zhang P. Fan Y. Ru H. Wang L. Magupalli V. G. Taylor S. S. (2019b). Crystal structure of the WD40 domain dimer of LRRK2. Proc. Natl. Acad. Sci. 116, 1579–1584. 10.1073/pnas.1817889116 30635421 PMC6358694

[B157] Zhang S. Xu W. Wang H. Cao M. Li M. Zhao J. (2019c). Inhibition of CREB-mediated ZO-1 and activation of NF-κB-induced IL-6 by colonic epithelial MCT4 destroys intestinal barrier function. Cell Prolif. 52, e12673. 10.1111/cpr.12673 31418947 PMC6869122

[B158] Zhang S.-J. Lin Y.-M. Zhou H.-C. Wei S.-D. Lin G.-H. Ye G.-F. (2010). Antioxidant tannins from stem bark and fine root of *Casuarina equisetifolia* . Molecules 15, 5658–5670. 10.3390/molecules15085658 20714319 PMC6257733

[B159] Zhao H.-Y. Fan M.-X. Wu X. Wang H.-J. Yang J. Si N. (2013). Chemical profiling of the Chinese herb formula Xiao-Cheng-Qi decoction using liquid chromatography coupled with electrospray ionization mass spectrometry. J. Chromatogr. Sci. 51, 273–285. 10.1093/chromsci/bms138 22977122

[B160] Zhong C. Mansour S. Nambiar-Veetil M. Bogusz D. Franche C. (2013). *Casuarina glauca*: a model tree for basic research in actinorhizal symbiosis. J. Biosci. 38, 815–823. 10.1007/s12038-013-9370-3 24287661

[B161] Zhong Y. Tu Y. Ma Q. Chen L. Zhang W. Lu X. (2023). Curcumin alleviates experimental colitis in mice by suppressing necroptosis of intestinal epithelial cells. Front. Pharmacol. 14, 1170637. 10.3389/fphar.2023.1170637 37089942 PMC10119427

[B162] Zhou P. Q. Fan H. Hu H. Tang Q. Liu X. X. Zhang L. J. (2014). Role of DOR-β-arrestin1-Bcl2 signal transduction pathway and intervention effects of oxymatrine in ulcerative colitis. J. Huazhong Univ. Sci. Technol. Med. Sci. 34, 815–820. 10.1007/s11596-014-1358-1 25480575

[B163] Zhu Y. Yang S. Zhao N. Liu C. Zhang F. Guo Y. (2021). CXCL8 chemokine in ulcerative colitis. Biomed. Pharmacother. 138, 111427. 10.1016/j.biopha.2021.111427 33706134

