# Supplementary figures and images for: Casuarina glauca branchlets’ extract as a potential treatment for ulcerative colitis: chemical composition, in silico and in vivo studies
Source: Front Pharmacol. 2023 Dec 22;14:1322181. doi: 10.3389/fphar.2023.1322181 (PMC10774231; doi:10.3389/fphar.2023.1322181)

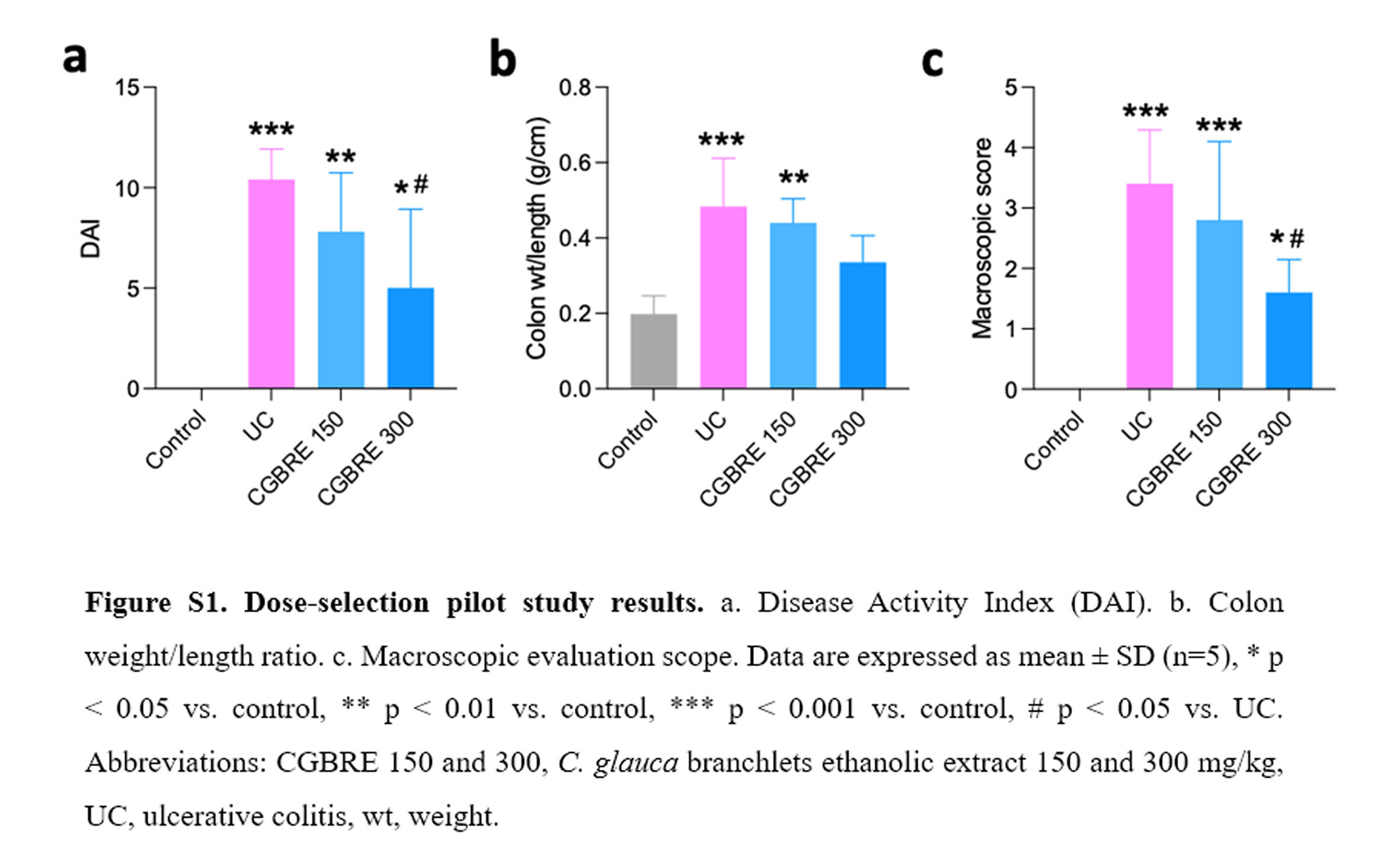

Supplement: Supplementary file 8 [file Image1.jpg]
